# Sediment Resuspension as a System-Wide Driver of Legacy and Bioavailable Phosphorus Release in Lake Erie

**DOI:** 10.1021/acs.est.5c17601

**Published:** 2026-04-10

**Authors:** Anshula Dhiman, Trevor Holm, Kendra Herweck, Audrey Ciochetto, Timothy Wahl, Fasong Yuan, J. Val Klump, Brice K. Grunert

**Affiliations:** † Biological Geological and Environmental Sciences, 2564 Cleveland State University, Cleveland, Ohio 44115, United States; ‡ Graduate School of Oceanography, University of Rhode Island, Narragansett, Rhode Island 02882, United States; § School of Freshwater Sciences, 14751 University of Wisconsin-Milwaukee, Milwaukee, Wisconsin 53204, United States

**Keywords:** internal phosphorus loading, legacy nutrients, sediment resuspension, benthic biogeochemistry, aquatic biogeochemistry, remote sensing

## Abstract

Freshwater ecosystems worldwide face persistent eutrophication and harmful algal blooms (HABs), driven primarily by external phosphorus (P) inputs from agricultural runoff. However, internal P loading remains poorly quantified in eutrophic systems such as Lake Erie, which store substantial legacy P pools within benthic sediments. Here, we resolved system-wide internal P loading from sediment resuspension, an overlooked but significant P cycling pathway. During the observed event, sediments released 2.3–11 × 10^–2^ g m^–2^ of bioavailable P, 22–256× greater than previously reported aerobic diffusive fluxes, highlighting resuspension as a major episodic internal P source in western Lake Erie. Using Sentinel-3 remote sensing reflectance, we quantified changes in suspended particulate matter (SPM) (ΔSPM) with a single-wavelength semiempirical algorithm that enabled a mechanistic, spatially resolved framework linking benthic sediment traits to satellite-derived SPM for basin-wide P release estimates during resuspension. Beryllium-7 analysis showed that repeated sediment mixing reworks multiyear deposits and remobilizes legacy P when resuspended. Compared to Maumee River spring P targets, this single event contributed ∼4.8% and ∼7.0% to total and soluble reactive P, respectively. Quantifying resuspension-driven P loads is expected to better constrain interannual HAB variability and contribute to assessing nutrient management outcomes for Lake Erie and similar aquatic systems.

## Graphical abstract

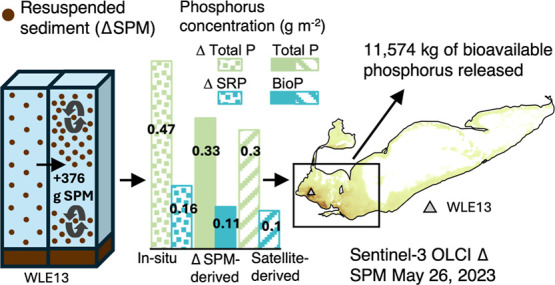

## 1 Introduction

Earth’s natural biogeochemical processes are interlinked, and this coupling across aquatic, terrestrial, and atmospheric systems is increasingly reshaped by anthropogenic influences.^1,2^ Lake Erie exemplifies this transformation of natural system functioning, as intensive agriculture, climatic variability, and invasive species contribute to the eutrophication of Lake Erie, leading to severe harmful algal blooms (HABs)^3^ and increased hypoxia in the central basin.^4^ In freshwater ecosystems, land-use change is often the most substantial contributing factor to eutrophication. Point (e.g., industrial) and nonpoint (e.g., agricultural) sources elevate the concentration of phosphorus (P), a key limiting nutrient, altering nutrient stoichiometry and shifting ecosystem dynamics toward cyanobacterial blooms (e.g., *Microcystis* in western Lake Erie, WLE).^5–10^ Current understanding of eutrophication is mostly derived from more easily observed external P inputs from tributaries,^3,5,11^ while the complex array of processes associated with internal phosphorus cycling remains underobserved.^12–17^

The reintroduction of legacy phosphorus (i.e., phosphorus accumulated in lake sediments from historical nutrient inputs) into the water column via internal pathways can significantly influence the overall phosphorus budget in shallow, eutrophic freshwater systems.^18,19^ However, quantifying internal P loading magnitude at the ecosystem scale has remained elusive, with observations spatially limited to small-lake studies and sediment incubations, and whole system studies limited to nutrient models.^14,20–22^ For large aquatic systems such as Lake Erie, the diffusive flux is treated as the primary internal phosphorus loading term based on relatively steady states of P release under oxic and anoxic conditions.^23–25^ Episodic loading from sediment resuspension has not been discretely characterized in these systems, despite the potential of these events to significantly elevate internal P loading and net sediment contribution to water column P, even relative to tributary loading.^18,26^

In Lake Erie’s western basin, the Maumee River delivers approximately 2.7 × 10^6^ kg of phosphorus annually from agriculture covering 72% of the watershed, including >70% of cropland using tile drainage that rapidly transports excess nutrients to tributaries.^11,27,28^ This external loading drives first-order variability and severity in annual HABs,^27,29,30^ and regulatory efforts have established “spring P target loads” to reduce these tributary inputs.^31^ However, considerable variability in bloom extent and severity remains unexplained by tributary inputs alone, particularly during severe HAB years,^3,30^ with internal phosphorus loading emerging as the largest unknown factor^32^ that could play a significant role in observed bloom dynamics.^15,17,24,33,34^ This internal cycling involves processes such as diffusive flux,^35^ biological excretion,^36^ and sediment resuspension-driven by storms and wind-induced mixing,^14^ and has been shown to mute or delay the ecosystem response to changes in external nutrient inputs via ‘ecosystem memory’.^19,34,37^

Diffusive fluxes have received the most attention due to more accessible rate measurement methods,^24,35^ while wind-driven resuspension and bioturbation remain less studied, despite their significant contributions to ambient phosphorus concentrations, as they are harder to constrain and quantify.^18,38^ In Lake Erie, wind-driven sediment resuspension can be well observed by satellite sensors using established suspended particulate matter (SPM) algorithms,^39^ where physical sediment mixing releases phosphorus from sediment pore water and loosely bound sediment fractions into the water column. This material is likely to become bioavailable and can be rapidly integrated into biomass.^40–42^ Moreover, as severe weather events intensify with shifting weather patterns, sediment resuspension events are expected to increase,^43^ amplifying internal phosphorus loading, and potentially shifting water quality improvements despite nutrient management efforts.^19,44^

This study characterizes sediment phosphorus biogeochemistry and deposition history from in situ observations and scales these insights using satellite observations to quantify the role of sediment resuspension in phosphorus cycling across western Lake Erie. We address: (1) what is the spatiotemporal history of P deposition, and what sediment layers actively exchange with the water column during resuspension events? and (2) what is the spatiotemporal P content of sediment, and what portions of the P pool are likely to be released and bioavailable during sediment resuspension events? We quantified sediment phosphorus fractions in the upper 5 cm along a gradient of Maumee River influence, characterized physical reworking of sediments using beryllium-7 (^7^Be) with a half-life (*T*_1/2_) of ∼ 53 days, and compared estimations of total and bioavailable phosphorus (TP and BioP) from sediment to in situ water column observations during a May 2023 resuspension event. Finally, we applied a validated SPM algorithm to satellite observations to provide event-scale estimates of the phosphorus loading. Through this approach, we offer a comprehensive assessment of internal P loading from sediment resuspension and its implications for phosphorus cycling, water quality, and nutrient management in Lake Erie and similarly impacted freshwater systems.

## 2 Materials and Methods

### 2.1 Study Site

The western basin of Lake Erie (Figure 1) is the shallowest portion of the lake (mean depth ∼ 7.5 m, max ∼ 19 m), receiving major inflows from the Detroit, Maumee, and Raisin Rivers.^11,45^ The Detroit River supplies ∼80% of water input from the upper Great Lakes, while the Maumee River, draining ∼17,000 km^2^ of agricultural land,^3,11^ carries >1.2 × 10^9^ kg of sediment annually and delivered spring TP and soluble reactive phosphorus (SRP) inputs of 1.1–2.0 × 10^6^ kg and 2.2–4.0 × 10^5^ kg (∼20% of TP), respectively, during 2017–2021.^45,46^ The peak discharge from spring snowmelt and rain fuels extensive algal blooms during summer, when the western basin is generally well-mixed with only occasional stratification.^31,32,43^ Prevailing southwesterly winds, shallow depth, and broad fetch of the western basin result in frequent resuspension and redistribution of fine bottom sediments.^26,43^

**Figure 1:**
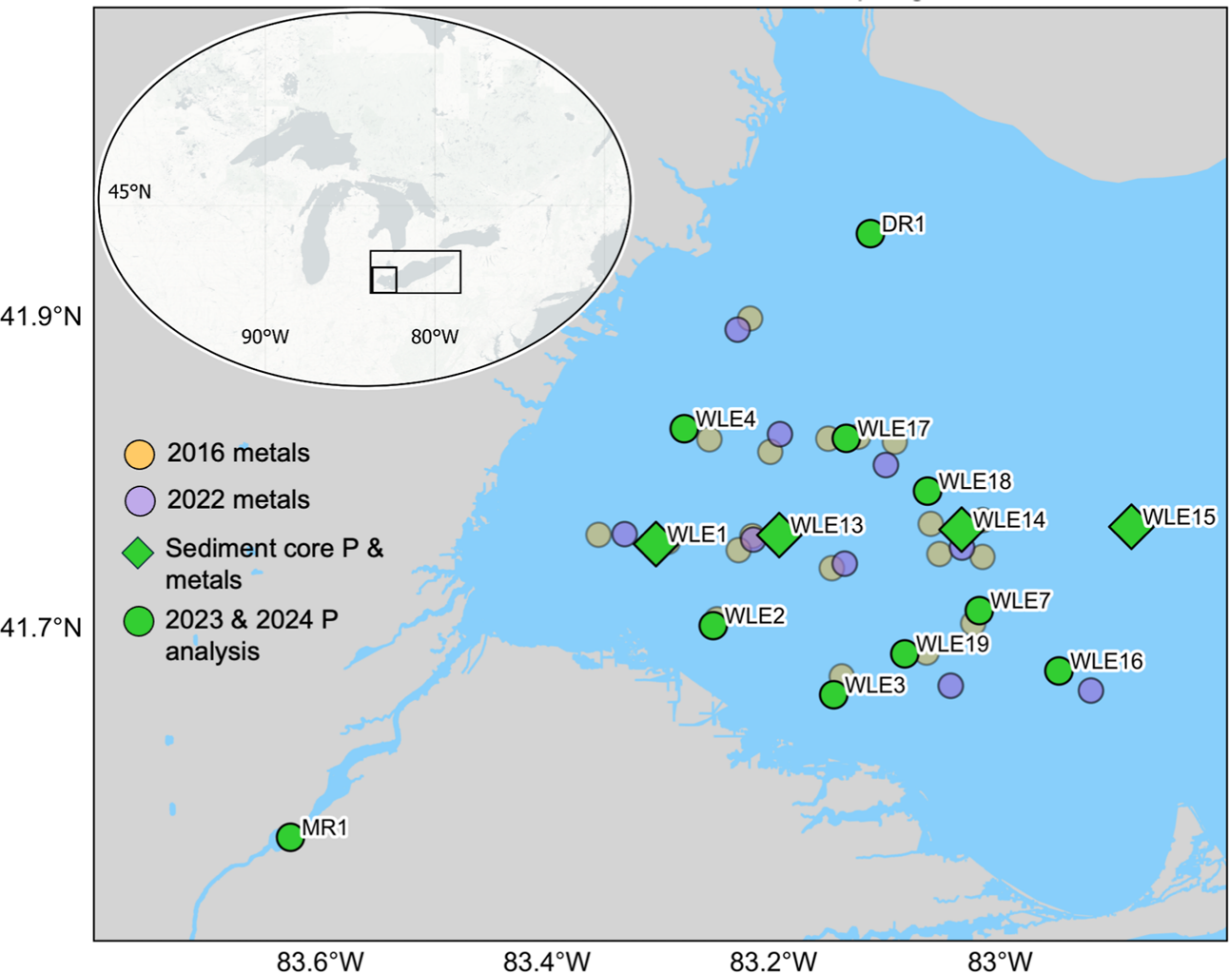
Western Lake Erie showing sampling sites for the water column and sediment parameters with a reference map as an inset.

### 2.2 Sample Collection and Preparation

Surface sediments (*n* = 12) were collected in May and August 2022 by using a Ponar grab. Sediment cores were collected using a GMX-25 Gomex box corer (Ocean Instruments) at stations WLE1, WLE13, WLE14, and WLE15 in April–June 2023, and April, June, and August 2024 (excluding WLE13; Figure 1). At each station, 4–6 polycarbonate cores were sectioned onboard at 1 cm intervals to 5 cm depth, and replicates were composited. Sediments were dried at 60 °C (∼72 h), homogenized, and sieved to ≤63 μm before phosphorus fractionation and metal analysis to remove coarse sand and mussel shell fragments.

Surface water samples (1 m depth) were collected at all stations (Figure 1) in 2023–2024 for TP, SRP, and SPM. SRP samples were immediately filtered (0.45 μm nylon filters) and acidified to pH < 2 (5.5 M H_2_SO_4_). Whole-water samples for TP were acidified immediately and refrigerated. SPM samples were collected by filtering water through precombusted (450 °C, 4 h) and preweighed glass fiber filters (0.7 μm) and stored at −20 °C. For SPM ^7^Be analysis, ∼300–800 L of water per site was filtered through a 0.45 μm cartridge filter using a high-volume pump and frozen until analysis. After resuspension, WLE13, WLE14, and WLE16 were resampled to detect changes in water column TP, SRP, and SPM.

### 2.3 Sample Processing and Analysis

Detailed sample processing and analytical procedures are provided in the Supporting Information (Section 2). Key methods are summarized below. Total phosphorus (particulate + dissolved P) in water was determined by digesting samples within 2 weeks of collection following EPA Method 365.1 ^47^ using a 50 mL aliquot of the sample with 1 mL of 5.5 M H_2_SO_4_ and 0.4 g of (NH_4_)_2_S_2_O_8_. SPM filters were dried at 105 °C for 24 h and reweighed to determine SPM concentration (Supporting Information Methods Section 2.1).^48–50^ High-volume cartridge filters were dried at 60 °C, retrieved from the outer casing, and placed in Marinelli containers for ^7^Be analysis.

Metals from sediment were extracted using USEPA Method 3050B^51^ (described in Supporting Information Methods Section 2.2), and metal data for 2016 were obtained from Yuan et al.^52^ for comparison with recent observations (2022–2023). Phosphorus fractionation for 2023 core samples followed a modified five-step sequential extraction^53^ using NaCl to extract loosely bound P, sodium bicarbonate-dithionite (NaBD) for redox-sensitive P, NaOH for Al/Fe oxide-bound P, HCl for Ca-bound P, and persulfate digestion for residual organic P (described in Supporting Information Methods Section 2.2). We define BioP as the labile fraction of TP, including loosely bound, redox-sensitive, and Al/Fe oxide-bound P fractions, that can be released during resuspension and contribute to dissolved SRP.^54^ SRP is the immediately bioavailable form of P measured in a water column.

Water column SRP, sediment-extracted P, and digested TP were measured (mg P L^–1^) as phosphorus using a Seal AQ2+ Automated Discrete Analyzer following EPA Methods 118 A and 134 A, respectively at Cleveland State University,^55,56^ with TP and SRP from 2023 independently analyzed at University of Wisconsin-Milwaukee’s School of Freshwater Sciences for quality assurance. Metals were analyzed via inductively coupled plasma mass spectrometry (ICP–MS). ^7^Be activity was determined using either an EG&G Ortec lithium-drifted germanium detector or an intrinsic germanium detector, coupled to a multichannel analyzer in a shielded room and calibrated using spiked sediment samples.^57,58^

Samples were analyzed in duplicate or triplicate, with accuracy verified using certified reference materials, including BCR-684 for sediment P fractionation, spiked samples, and independent reference standards (RICCA, 1 mg P L^–1^ and DIONEX, diluted to 1 mg P L^–1^) for AQ2+. Spike recoveries for AQ2+ ranged from 85% to 105%.

### 2.4 Satellite SPM Estimation

We used the full-resolution (300 m) Level 1B Top-Of-Atmosphere (TOA) radiance product from the European Space Agency’s Sentinel-3A/B Ocean and Land Color Instrument (OLCI) from NASA’s Atmosphere Archive and Distribution System Distributed Active Archive Center (LAADS.DAAC)^59^ to quantify SPM and phosphorus in western Lake Erie. The Level-1B data were atmospherically corrected using POLYMER in optically complex inland waters,^60^ and retrieved water-leaving reflectance (ρ_*w*_ = π*R*_*rs*_) at 665 nm was then used to estimate SPM (Figure 2a,b) using eq 14 from Nechad et al.^39^

**Figure 2:**
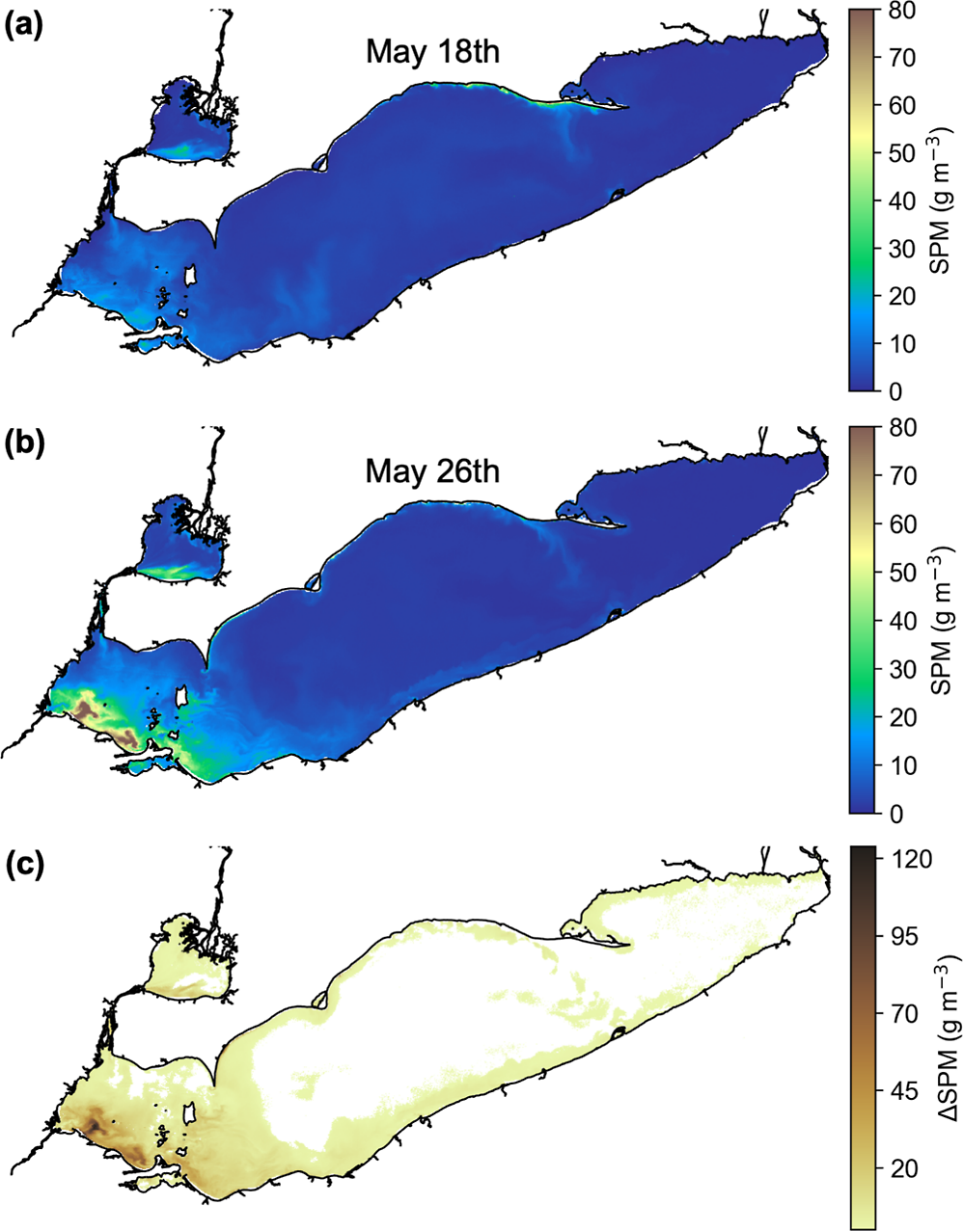
Satellite-observed changes in suspended particulate matter (ΔSPM) across Lake Erie. SPM distributions were derived from POLYMER atmospherically corrected Sentinel-3A/B OLCI reflectance at 665 nm using the Nechad et al.^39^ algorithm. Panels (a,b) show SPM concentrations (g m^–3^) on May 18 and May 26, 2023, and panel (c) shows the change in SPM (ΔSPM, g m^–3^) between these dates across Lake Erie for pixels where the relative change in SPM was greater than the algorithm performance threshold (*NRMSE* ≤ 13.9%).

The algorithm performance was validated using in situ SPM and radiometry collected from 117 sites across the Great Lakes, including 78 sites in Lake Erie (2023–2024). In situ reflectance was measured using a hand-held SpectraVista Corporation HR-512i spectroradiometer following methods outlined by Mobley et al.^61^ and the IOCCG protocol.^62^ Surface effects were corrected using the approach of Groetsch et al.^63^ Sediment resuspension pixels were identified from difference in satellite-derived SPM (ΔSPM) between May 26, 2023 (observed resuspension event) and the closest clear sky preresuspension image on May 18, 2023 (Figure 2), agreeing well with in situ measurements from May 23–24, 2023. Pixels were classified as resuspended when the percent change in SPM exceeded the SPM algorithm uncertainty threshold [13.9%, expressed as root-mean-square error (*NRMSE*) normalized by the range of observed SPM concentration; Figure S1].

### 2.5 Spatial and Temporal Variability of Sediment Phosphorus Fractions

Using one-way ANOVA, we assessed temporal variation within each station by comparing P fractions across months (April-June) with sample sizes ranging from *n* = 10 to 20. Spatial variations were evaluated by comparing P concentrations among core stations within each month (*n* = 20). Post-hoc pairwise comparisons for specific months and station pairs were performed using Tukey HSD (α = 0.05). The spatial coefficient of variation was calculated as the standard deviation of the P fraction concentration divided by its mean across stations for each month.

### 2.6 Calculations and Estimates

To estimate the rates at which particles are redistributed within the upper sediment column, we calculated sediment vertical mixing rates (*D*_*b*_) by fitting eq 1 ^64^ to each sediment ^7^Be profile (Figure S2a,b) for 2023

A(dpmcm−2)=ATexp(−(λDb×z)) 1

where *A* is ^7^Be activity (dpm cm^–2^) at depth *z* (cm), *A*_*T*_ is surface activity of a well-mixed layer (0–1 cm; dpm cm^–2^), λ is the radioactive decay coefficient (days^–1^) (calculated as ln(2)/*T*_1/2_), and *D*_*b*_ is the mixing coefficient (cm^2^ y^–1^), assumed to be constant over 0–5 cm.^64^ Mixing depths (*h*) were calculated as the depth over which ^7^Be-tagged particles could redistribute by mixing at the estimated *D*_*b*_ during the tracer’s average life (τ ∼ 77 days)^57,65^

2 $$h\left(cm\right)=\sqrt{D_{b}\tau }$$

Using ^7^Be total inventories, i.e., the sum of ^7^Be activity at all sediment depths (*I*_*sed*_), the steady state flux (*J*_*sed*_) was calculated as

3 $$J_{sed}\left(dpm{cm}^{-2}{day}^{-1}\right)=\lambda \times I_{sed}$$

This flux represents the ^7^Be-bearing particle deposition rate. As ^7^Be attaches rapidly to suspended particles,^64^ water column particle residence time (*T*_res_) was calculated using SPM ^7^Be activity (*A*_w_, dpm m^–3^), *J*_sed,_ and water column depth (as *D*) as

4 $$T_{res}\left(days\right)=\frac{A_{w}\times D}{J_{sed}}$$

Using particle residence time, settling rates of particles were also calculated as

5 $$settling\;rate\;\left(m{days}^{-1}\right)=\frac{D}{T_{res}}$$

The time-averaged ^7^Be depositional flux (*J*_Be_, dpm cm^–2^) from inventory changes between sampling dates after correcting for radioactive decay

6 $$J_{Be}\left(dpmcm^{-2}day^{-1}\right)=\frac{\lambda \left[I_{2}-I_{1}\exp\left(-\lambda t\right)\right]}{1-\exp\left(-\lambda t\right)}$$

where *I*_*1*_ and *I*_*2*_ are ^7^Be inventory at successive sampling month (dpm cm^–2^), and *t* is the time between the two sampling dates (days). The sediment mass accumulation rate was then estimated by converting this ^7^Be flux to sediment flux using measured mass-specific ^7^Be activity of SPM (*A*_spm_) (dpm g^–1^) as

$$accumulation\;rate\;\left(AR\right)\;(g{cm}^{-2}{year}^{-1})=\frac{J_{Be}}{A_{spm}}$$ 7

Dry bulk density (ρ_b_, g cm^–3^) and porosity (ϕ) were calculated from sediment dry mass, assuming particle density (ρ_d_) of 2.45 g cm^–3^.^66^ Erosion depths from resuspension were estimated using equation adapted from Niu et al.^67^

8 $$erosion\;depth\;\left(ED\right)\left(cm\right)=\frac{\left({SPM}_{after}-{SPM}_{before}\right)\times D}{\rho_{b}\times \left(1-\phi \right)}$$

where *SPM*_before_ and *SPM*_after_ represent SPM concentrations before and after resuspension (g m^–3^). The product of this in situ change in SPM (ΔSPM) and *D* gives the mass of sediment resuspended (*M*_resuspended_, g m^–2^) at each coring station (Figure 1). Similarly, changes in water column TP (Δ*TP*_water,_ g m^–2^) and SRP (ΔSRP_water_, g m^–2^) were estimated over the water column depth. For a realistic estimate of P associated with resuspended bed sediment, thickness-weighted average P concentration (*P*_sediment,_ g m^–2^) was calculated over the estimated eroded layer (from eq 8), accounting for depth-specific P concentration and partial erosion of the deepest layer ( $P_{n}\left(ED-\sum_{i=1}^{n-1}\Delta z_{i}\right)$ ). This P concentration, averaged over the erosion depth, was then multiplied by *M*_resuspended_ to estimate TP mobilized and BioP released (*TP*_sediment_ and *BioP*_sediment_) during resuspension

$$P_{se\dim ent}\left({gm}^{-2}\right)=\frac{\overset{n-1}{\underset{i=1}{\sum }}\left(P_{i}{\Delta z}_{i}\right)+P_{n}\left(ED-\overset{n-1}{\underset{i=1}{\sum }}{\Delta z}_{i}\right)}{ED}\times M_{resuspended}$$ 9

where *P*_*i*_ is the TP or BioP concentration in sediment layer *i* (mg g^–1^), Δ*z*_*i*_ is the thickness of layer *i* (cm), and *n* is the index of the layer that is partially eroded (the last layer reached by erosion depth). Sediment P quantifications were also scaled to satellite observations using

$$P_{\mathrm{scaled}}\left({gm}^{-3}\right)={\mathrm{P̅}}_{\mathrm{sediment}}\times \Delta SPM$$ 10

where *P*_scaled_ represents satellite-derived internal loading (*TP*_scaled_ and *BioP*_scaled_) using *P̅*_sediment_ that is the mean bed sediment concentration (of ${\overset{®}{TP}}_{\mathrm{sediment}}$ and ${\overset{®}{BioP}}_{\mathrm{sediment}}$ , g m^–3^) averaged across sampled depths, stations, and months in 2023, and the satellite-derived ΔSPM, providing first-order system-wide mechanistic quantification of internal P loading. Satellite-based site-specific P loads (*TP*_satellite_ and *BioP*_satellite_, g m^–2^) were calculated using eq 9, with *M*_resuspended_ and *ED* derived from satellite-based ΔSPM from a single pixel colocated with each sampling site and corresponding pixel-specific water depth from the NOAA bathymetry data set.^68^

## 3 Results

### 3.1 Dynamic Sediment Processes and Their Implication for Phosphorus Cycling

In dynamic systems such as Lake Erie, the short-lived ^7^Be radionuclide (*T*_1/2_ ∼ 53 days; average life of ∼77 days) binds to fine particles and decays on time scales comparable to short-term depositional processes, thus typically confined to surface sediments. However, its detection below the uppermost layer in sediment cores indicated a vertical redistribution of recently deposited material by sediment mixing (Figure 3). Sediment resuspension or dilution from older ^7^Be-depleted deposits can reduce the intensity of surface signals relative to the underlying sediments. Among shallower nearshore sites (relative to the Maumee River), WLE13 showed overall lower but more variable ^7^Be inventories, including periods of minimal activity in May 2023 (Figure 3a). Coarser sediment (lower porosity, ∼63–89%) and mussel shells observed at this site indicated frequent resuspension and preferential removal and transport of finer, ^7^Be-bearing particles,^57,64,66^ resulting in limited deposition of new material, also evident from lower mass-specific SPM ^7^Be activity (6.62 dpm g^–1^; Table S1). Station WLE1, near the Maumee River mouth, displayed higher and relatively constant ^7^Be activities to depths of 2–3 cm (Figure 3a), reflecting a balance between river-derived deposition of freshly tagged material and its frequent resuspension. Also, a shorter fetch under the prevailing southwesterly wind climatology likely reduces the frequency and intensity of sediment resuspension and dispersal compared to WLE13, promoting sediment accretion. Offshore stations, WLE14 and WLE15, generally exhibited higher ^7^Be activities than nearshore sites with signal reaching up to 4–5 cm (Figure 3a,b), indicating deposition and vertical mixing of ^7^Be-tagged particles into deeper layers. Higher proportions of fine-grained, dark-colored sediment with higher organic content, and porosity (up to 92% at WLE15) further supported their characterization as focusing/depositional zones in western Lake Erie.^66^ These results are consistent with observed spatial patterns in SPM variability from satellite, showing higher SPM concentrations at shallow nearshore sites, indicating greater dispersal from these sites (Figure 2b,c).^39^

**Figure 3:**
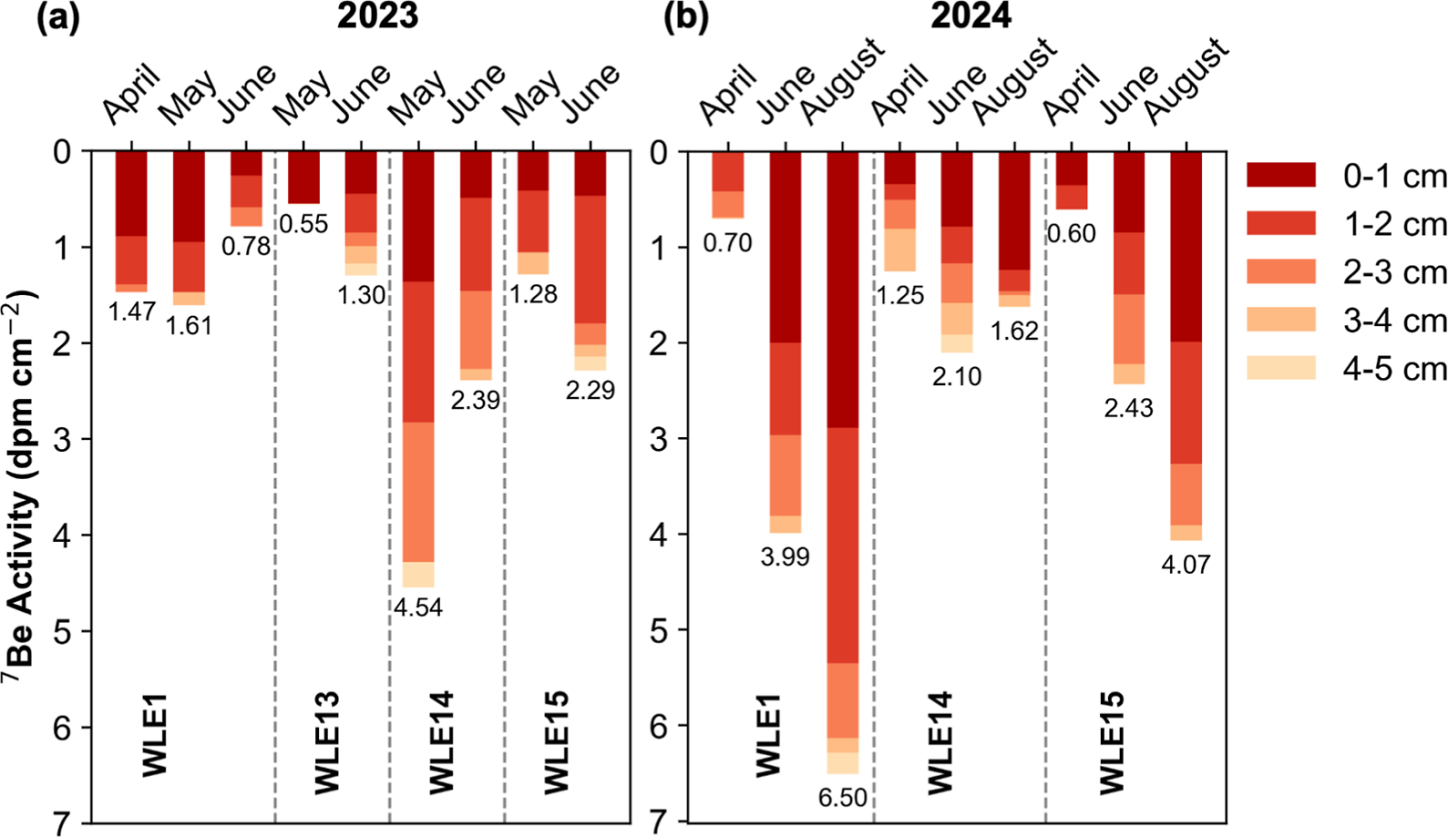
Stacked bar plots showing depth-integrated ^7^Be activity profiles across Lake Erie core sites at 1 cm depth intervals (0–1, 1–2, 2–3, 3–4, and 4–5 cm) for (a) 2023 and (b) 2024, highlighting temporal changes in observed penetration depth and inventories. Values below each bar denote the total ^7^Be inventory (dpm cm^–2^) for that sediment core.

To further understand the sediment dynamics, we estimated vertical mixing rates, mixing depth, particle residence time, settling rates, and accumulation rates (using eqs 1–7) based on the ^7^Be inventories in sediment and SPM. Sediment resuspension increases these rates by physically disturbing and reworking sediments at higher intensity, promoting deeper mixing (higher mixing depths), as observed in WLE14 and WLE15. Such events also increase particle residence time in the water column and reduce settling rates, while a short residence time indicates rapid settling.^57^ At nearshore WLE1, lower vertical mixing rates (*D*_*b*_) showed relatively calm periods (14.5 to 29.8 cm^2^ yr^–1^; Figures S2 and S3) that promoted accumulation of freshly ^7^Be-tagged material and occasional homogenization of sediment depths up to ∼3–5 cm (April and May 2023; June and August 2024; Figure 3a,b). During spring 2023, between April and June, the mixing rates increased at WLE1 (from 14.5 to 70.51 cm^2^ yr^–1^; ≈5×) and WLE13 (from 5.43 to 69.6 cm^2^ yr^–1^; ≈12×), and surface ^7^Be activities (0–1 cm) decreased below subsurface values, indicating erosion of surface sediments from intensified weather conditions (Figure 3a). Particle residence time at WLE1 extending to ∼ 11 days (from 1–2 days; Table S2) and decreased mass-specific ^7^Be activity of SPM (from 18.58 to 7.15 dpm g^–1^; Table S1) showed mobilization of older bed material rather than newly delivered particles. The nearshore reductions in ^7^Be inventories coincided with higher offshore inventories at WLE14 (Figure 3a), and elevated mass-specific SPM activities at both WLE14 and WLE15 (39.9 and 26.6 dpm g^–1^) support offshore transport and accumulation of fine, freshly ^7^Be-tagged particles despite episodic resuspension.

Offshore stations also exhibited higher particle settling rates and shorter residence time (1.9–4.7 m d^–1^ offshore vs 0.5–2.3 m d^–1^ nearshore; Table S2), favoring efficient deposition of young particles, and resulting in lower ^7^Be-tagged suspended loads (0.03–0.14 dpm L^–1^) compared to the shallower sites (0.21–0.24 dpm L^–1^). Depositional zones also showed a greater estimated sediment accumulation rate (3.2 g cm^–2^ yr^–1^; eq 7), deeper ^7^Be penetration, and mixing depths (Figure 3a,b and Table S3). These sites experienced occasional episodic erosion as well, including during the observed May 2023 resuspension event and evidently from June 2023 dynamics showing increased mixing rates (up to ∼200 cm^2^ yr^–1^) and reduced surface (0–1 cm) ^7^Be activities below subsurface levels (Figure 3a), similar to nearshore sites. This was further supported by ^7^Be inventories at WLE14 declining faster than radioactive decay between May and June 2023 (Figure S4a) due to the removal of ^7^Be-tagged particles. In 2024, mixing rates declined toward August (Figure S3), while ^7^Be inventories increased over the same interval (Figure 3b), highlighting a trend toward more stable deposition consistent with prevailing wind climatology for this region.^26,43^

Resuspension and mixing of benthic sediment dictate whether sediment-bound P is stored or remobilized. Our ^7^Be results, together with sediment characteristics and lower mass accumulation rates at WLE1 and WLE13 (0.50 and 1.98 g cm^–2^ yr^–1^, respectively), indicated higher nearshore erosion, favoring dispersal of fine particles and associated legacy P toward offshore depositional sites. Because of periodic mixing and resuspension, nearshore and offshore depositional zones are not permanent archives of age-stratified external inputs, as mixing homogenizes upper layers and episodically resuspends material to the water column. This is consistent with vertical profiles of P fractions within the upper 0–5 cm, showing no significant differences in concentration (Figure 4a; *p* > 0.05; Table S4) and low variability in percent composition with depth (typically <3.5%). Stable metal distributions in these cores and across western Lake Erie from 2016-2023 also support these results (Table S5).^52^

**Figure 4:**
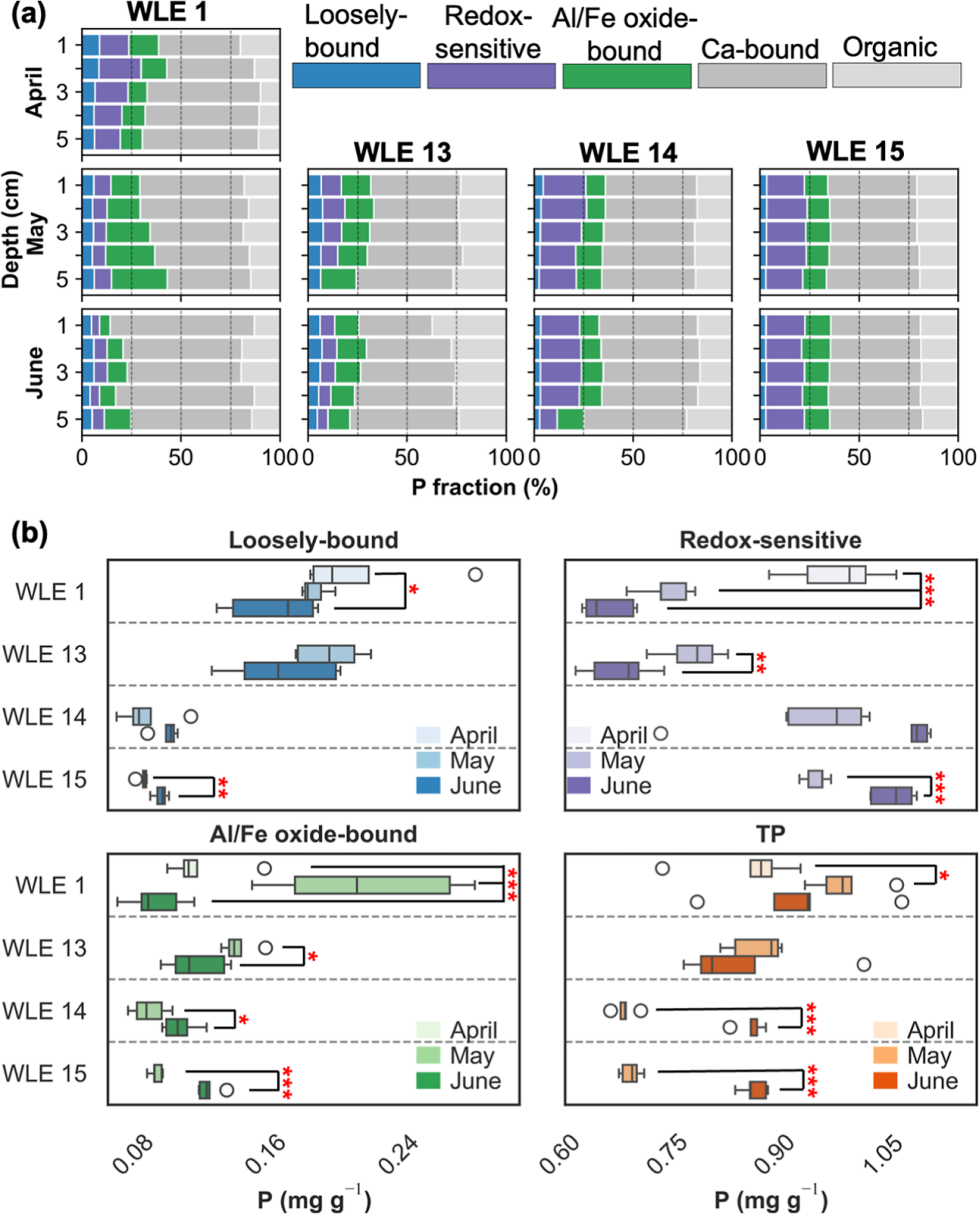
Sediment phosphorus fractions and their spatiotemporal variability in western Lake Erie sediments in 2023. Panels in (a) show relative depth profiles of P fractions (loosely bound, redox-sensitive, Al/Fe oxide-bound, Ca-bound, and organic) at 1–5 cm intervals for April, May, and June, at different core stations, and (b) panels show boxplots of absolute concentrations (mg g^–1^) for BioP fractions (loosely bound, redox-sensitive, and Al/Fe oxide-bound) and TP across stations. Monthly differences highlight shifts in bioavailable pools and TP, with statistical significance indicated (*p < 0.05, **p < 0.01, ***p < 0.001). Boxes show the interquartile range (*IQR*) with medians, whiskers represent 1.5 × *IQR*, and circular markers denote outliers.

Statistical ANOVA results revealed that most P fractions showed higher spatial (degree of freedom = 3 between stations, 16 within groups) variability in April and May of 2023, due to higher concentrations at WLE1 and WLE13 from the Maumee River spring loading (Figure 4b). By June, P fraction concentrations increased at offshore sites while decreasing at nearshore sites (Figure 4b), reducing the spatial coefficients of variation from ∼17–50% to 50% and from ∼8 to 35%. Redox-sensitive P was proportionally higher offshore due to preferential accumulation of fine-grained particles providing abundant Fe/Al oxyhydroxide surfaces for phosphate adsorption and deposition with settling organic matter.^40,69^ Overall, our ^7^Be and P data showed that legacy P is repeatedly reworked and mixed with newer inputs through resuspension, with nearshore erosion and offshore focusing controlling where P is stored and when it is remobilized, thereby influencing internal loading.

### 3.2 Mechanistic Understanding of Phosphorus Release during Sediment Resuspension

In western Lake Erie, resuspension events are episodic but influential, driving repeated cycles of erosion and redistribution before permanent burial of sediment and associated phosphorus (Figure 3).^52,57^ Typically, the water column TP scaled linearly with SPM (Figure S5a), showing a stronger first-order relationship across 2023 and 2024 (*R* ^*2*^ = 0.73 and *R* ^*2*^ = 0.66, respectively) than TP versus chlorophyll a (Figure S5b). The May 2023 resuspension event departed from this background regime, showing elevated particle concentrations but a lower TP content (Figure S5a). In a steady-state system with burial/export as the primary phosphorus loss pathway, we would expect to observe similar TP concentrations between the water column and benthic sediment. However, the lower TP/SPM ratio suggested significant recycling of TP from resuspended particles.^66^ Estimated settling rates indicated prolonged particle residence times in the water column (Table S2), highlighting P recycling through diffusive flux or sediment resuspension, and potentially exporting material to Lake Erie’s central basin.^26,43^ To evaluate this, we considered phosphorus release to the water column during an observed resuspension event.

In May 2023, water column conditions observed before and during sediment resuspension illustrated its significant role in mobilizing TP and releasing BioP to the water column (Figure 5a). The observed water column, sediment-derived, and satellite estimations of P mobilization from bed sediments during resuspension displayed spatiotemporal patterns, with WLE13 showing the highest increase in SPM, TP, SRP, and BioP (an order of magnitude higher; Figure 5). These observations were consistent with ^7^Be results, which identified WLE13 as the most frequently resuspended shallower site with a higher sediment P content in May 2023 (see Results Section 3.1). Thus, the magnitudes of SPM, TP, SRP, and BioP increased toward the Maumee River and decreased offshore, showing how differences in porosity and erosion depth drive variation in resuspended sediment and internal P loading across sites (Figure 2b,c, 5a,c, and 6).

**Figure 5:**
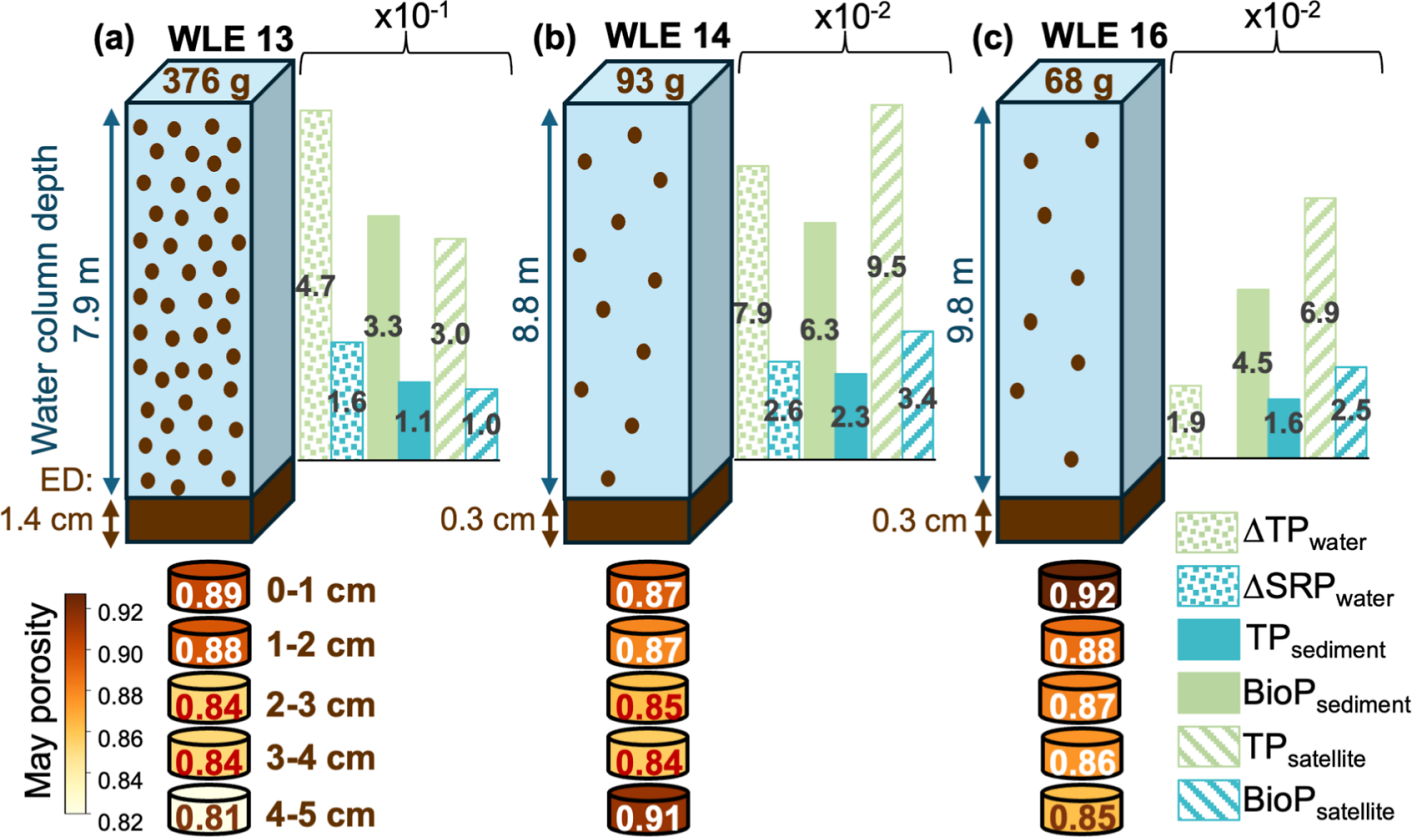
Conceptual representation of sediment resuspension and associated internal phosphorus loading in western Lake Erie based on the observed May 2023 resuspension event. Each panel shows the mass of in situ sediment resuspended (g), water column depth (m), and erosion depth (cm) for three sites observed during the sediment resuspension event: (a) WLE13, (b) WLE14, and (c) WLE16. Bar graphs compare observed water column change in TP and SRP (g m^–2^) (Δ*TP*_water_ and Δ*SRP*_water_ as dotted bars), *TP*_sediment_ and *BioP*_sediment_ as solid bars, and *TP*_satellite_ and *BioP*_satellite_ as striped bars. The P concentration for each estimate is integrated over erosion depth. Note that *y*-axis scales differ among panels, with values at WLE13 an order of magnitude higher than at the offshore sites. Each core section below the water column schematic shows measured porosity values across the upper 5 cm of sediment, with the color scale on the left corresponding to observed porosity in May.

The shallower nearshore WLE13 showed the highest erosion depth (1.39 cm; estimated using eq 8) and, along with elevated spring TP and BioP concentrations in sediments, displayed greater increases in TP and SRP relative to the sediment loaded to the water column [TP/SPM = 1.25, 0.86, and 0.29 mg g^–1^, and SRP/SPM = 0.43, 0.32, and <0.002 mg g^–1^ (SRP below detection) for WLE13, WLE14, and WLE16, respectively]. Offshore, WLE14 and WLE16 (representative of WLE15 based on observed sediment characteristics) exhibited smaller changes in SPM and water column P (Figure 5b,c). Despite similar erosion depths (∼0.3 cm) to WLE14, WLE16 did not display a comparable increase in SRP, likely due to its location on the periphery of the resuspension event, receiving water masses carrying resuspended material already depleted of BioP by the time of sampling. Annual wind climatology also suggests low sediment deposition at WLE16, as dominant southwest winds promote more northeastward deposition of P-rich material^67^ (Figure 6), i.e., near WLE14 and WLE15, highlighting the challenge of scaling first-order estimates of P loading across stations. Critically, our theoretical estimates based on lab-measured BioP fractions (using eq 9) agreed well with the observed water column increases in SRP (Figure 5a,c), indicating that BioP released from resuspended sediments contributed to the observed SRP pulse. These agreements were strongest at directly measured sites, WLE13 and WLE14, with general alignment when scaling WLE15 sediment traits to WLE16 observations.

**Figure 6:**
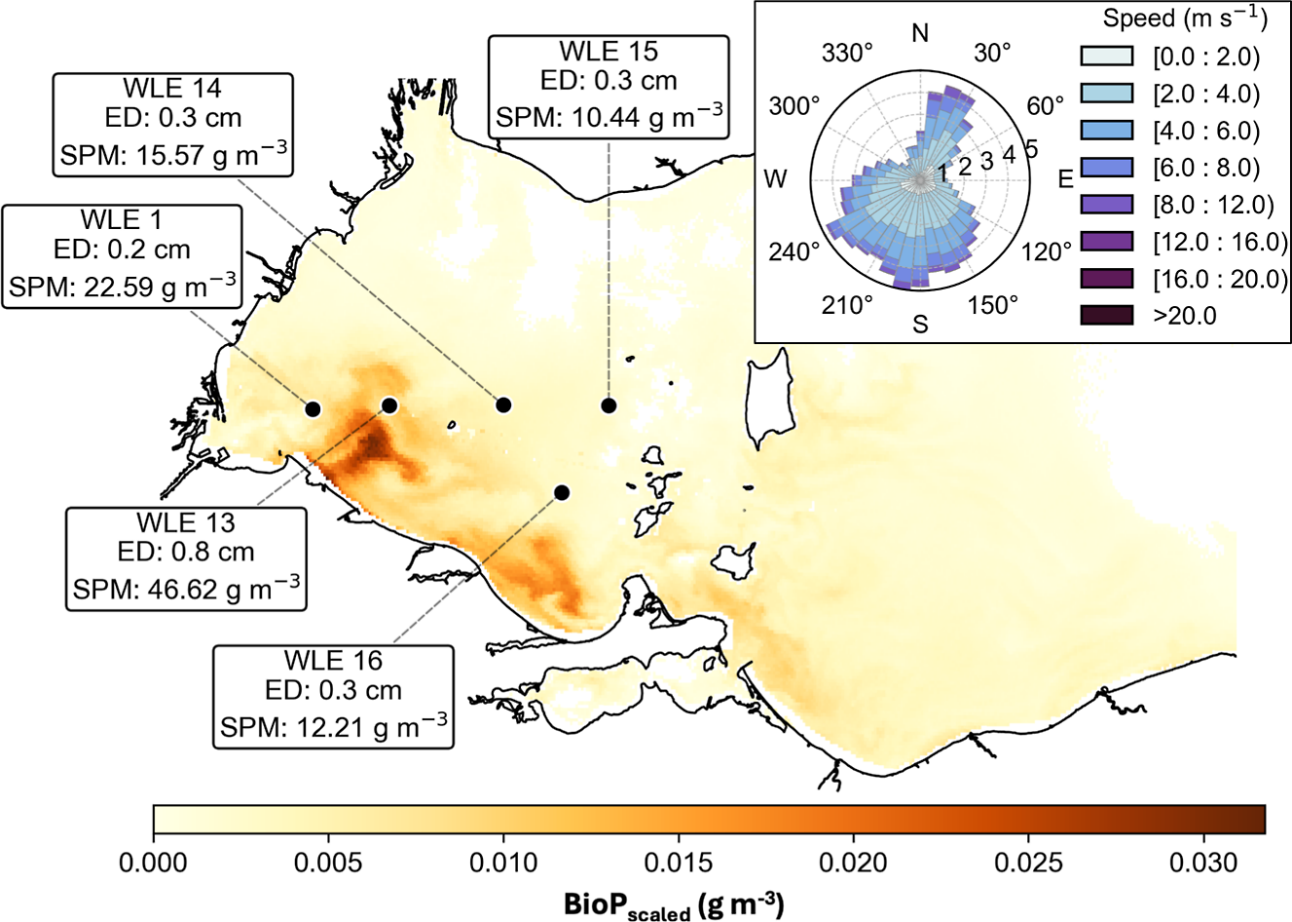
Event-scale mapping of satellite-derived bioavailable phosphorus (*BioP*_scaled_, g m^–3^) release from sediment during May 2023 resuspension in western Lake Erie. The map shows core sites and one noncore site (WLE16) with satellite-derived ΔSPM concentration (g m^–3^) and erosion depth (*ED*, cm) estimated from it and average porosity. Empty pixels represent areas where the percent change in SPM was below the SPM algorithm performance threshold (*NRMSE* ≤ 13.9%). The inset wind rose shows predominant southwesterly winds at NOAA’s National Data Buoy Center (NDBC) THRO1,^70^ with radial axes showing percentage frequency of wind directions and colors representing wind speed bins.

Based on general agreement between in situ, sediment, and satellite-derived estimates of erosion depth and P release (Figure 5), we quantified event-scale internal P loading (*BioP*_*scaled*_; Figure 6) for the May 2023 resuspension using ΔSPM derived from Sentinel-3A OLCI scenes on May 18 and 26 (Figure 2c). Satellite-derived SPM agreed well with in situ observations with a typical multispectral sensor performance (*NRMSE* ≤ 13.9%, Figure S1). Estimated bed sediment erosion depths from ΔSPM ranged from 0.2 to 0.8 cm for western Lake Erie (Figure 6), accounting for 89.2% of total resuspended sediment across the lake (Figure 2b,c). This corresponded to 40,314 kg of TP mobilized (expected to largely resettle with resuspended particles), of which 12,665 kg of BioP was released as dissolved phosphorus to the water column, mostly within the western basin (Figure 6). These values represent ∼1.6% and 3.3% of the 2017–2021 mean Maumee River spring TP and SRP loads, or ∼4.7% and ∼7.0% of the current spring target loads (TP = 86 × 10^4^ kg and SRP = 18.6 × 10^4^ kg), respectively,^45^ indicating the role of sediment resuspension events as large, episodic internal fluxes of P to the water column.

## 4 Discussion

Our ^7^Be results showed that short-lived, wind-induced disturbances continually rework recently deposited material, homogenizing new inputs with older deposits. As a result, the upper 0–5 cm behaves as actively mixed layers, explaining the benthic stability of the vertical distribution of externally sourced phosphorus across sediment cores in WLE (Figure 4a). Consistent with previous findings that showed no significant depth-dependent P variations,^71^ our results support that surface sediments act as an actively mixed reservoir of P that can be mobilized during resuspension to supply dissolved BioP to the overlying water column.^66^ Thus, recycling of P from bed sediments during episodic resuspension disrupts particle burial and increases the likelihood that P associated with this material is transported downstream (e.g., to Lake Erie’s central basin). Given typical reported sedimentation rates of ∼ 0.3–0.6 g cm^–2^ yr^–1^ for WLE^24,52,72,73^ and estimated mixing depths (*Z*_*mix*_, calculated using eq 2; Table S3), the actively mixed layer integrates deposition over years to decades that would otherwise be expected to stratify in the sediment record. Thus, a resuspension event reintroduces legacy P accumulated over multiple years back to the water column at levels reflecting the average P load over this temporal window. Further research is needed to determine the age of sediment overturned within the mixing zone to distinguish fresh deposits from legacy material and to characterize the depth at which P burial is constant and the extent to which P is lost from the active sediment layer.^66,74^

The active sediment mixing redistributes and laterally transports P-enriched Maumee River-derived material from nearshore sites toward offshore zones that serve as more efficient sediment retention regions under annual dominant southwest winds (Figure 6), leading to spatiotemporal homogenization of sediment P traits.^40,43,66^ Offshore locations also exhibited frequent resuspension events outside observations directly made here (e.g., June 2023 and April 2024). Together, these observations support an important contribution of sediment resuspension to the TP loading budget for a given year, impacting ecological functioning and HAB evolution during the growing season.^18,42^ Our opportunistic sampling coincides with satellite measurements, which highlights the utility of satellite-derived observations for quantifying the impact of benthic resuspension on the overall P budget. Episodic phosphorus inputs from these events are expected to play a significant role in explaining observed variability in severe HAB years.^75^

As an initial, scalable approach using eq 10, we used averaged sediment TP and BioP to quantify first-order event-scale internal P loading from sediment resuspension (Figures 2c and 6). While this introduces spatiotemporal uncertainty from spatial and monthly heterogeneity,^33^ over longer time scales (e.g., satellite time series), our approach suggests that this uncertainty will be relatively small. Across sites, estimated BioP release from sediment resuspension (2–11 × 10^–2^ g m^–2^ event^–1^: expressed as SRP equivalent release) was 22–256 times higher than the typical aerobic diffusive flux of 0.43–0.91 × 10^–3^ g m^–2^ day^–1^.^24^ This expected difference reflects that sediment resuspension physically entrains surface sediment layers, mobilizing a larger mass of TP and BioP from eroded sediment layers and porewaters than passive diffusive flux. Our ^7^Be data indicated that resuspension events of this magnitude occur ∼ monthly and penetrate to deeper depths than we directly observed (Figure 3 and Table S3). While constant diffusive flux of relatively low P concentrations has been reported to sustain but not initiate HABs on discrete time scales,^24^ the rapid introduction of large quantities of BioP during sediment resuspension has the potential to significantly modulate phytoplankton dynamics.

To date, nutrient management efforts in Lake Erie and similar systems have largely focused on external nutrient loads, with modeled lake response (e.g., HAB severity) tied to these loads.^25^ However, sediment resuspension reintroduces legacy P into the water column, shaping ecological and water quality outcomes alongside these external inputs.^18,19^ Our study provides the first event-resolved, basin-wide estimate of internal TP mobilization and BioP release from sediment resuspension in western Lake Erie.^26,71^ This single, moderate sediment resuspension event accounted for ∼4.8% (TP) and ∼7.0% (SRP) of the target spring loading from the Maumee River, the largest Great Lakes tributary. Although this is not “new” P, and the system may not elicit the same response as from external P inputs,^19,38^ our approach quantifies the extent to which episodic events modulate reductions in external inputs and influence contemporary ecological state. It also offers more holistic insights into potential ecological shifts under a changing climate^43,44^ and is critical to fully assess how ongoing, management-driven changes in external inputs are manifesting in intended water quality outcomes.

In Lake Erie and other similar lake environments, benthic sediments serve as substantial P reservoirs.^20,35,76,77^ Lakes such as Okeechobee, Peipsi, Võrtsjärv, Green Valley Lake, and Missisquoi Bay store large, labile sediment P pools that have been reported to, or have the potential to, resuspend or release P during sediment resuspension events, producing episodic phosphorus fluxes that can exceed external inputs.^20,78^ As in Lake Erie, these lakes integrate multiyear watershed processes into sediment layers that readily interact with the water column, shaping ecological outcomes. Despite acknowledging the significant role of internal processes in regulating when and where P becomes available and, in turn, ecological responses, most ecological and bloom-forecast models rely on external loading or only consider constant diffusive terms, due to challenges in constraining episodic events like sediment resuspension.^15,17,21^ Indeed, mass-balance modeling studies have accounted for sediment resuspension among other factors, but cannot define or quantify these episodic processes.^18^ Through targeted sampling of sediment traits and satellite imagery, our approach showed promise for quantifying P loading from sediment resuspension across these systems, in turn informing bloom-forecast models.^75^ This study showed that sediment resuspension not only mobilizes particulate TP but also releases significant BioP to the water column, which can be rapidly assimilated into biomass^1,33^ and is consistent with studies showing ∼20–30% of P delivered to freshwater systems is recycled and ultimately exported.^66^ Understanding these dynamics over longer timeframes can help quantify the role of internal P loading in bloom development and severity and improve accounting of the coupled dynamics between the western and central Lake Erie basins that fuel hypoxia.^4,79^ The framework developed here can be applied to quantify P release from sediment resuspension in Lake Erie and similar aquatic systems and provide data sets to support further investigations of the role of internal P loading from resuspension on ecological processes,^3,42^ including whole ecosystem response to ongoing nutrient management efforts.

## Supplementary Material



## Data Availability

In situ and remote-sensing observations are openly accessible through NASA’s SeaBASS data repository under PI Brice Grunert. All code for analysis and related data are also publicly available in the public repository on GitHub (https://github.com/Carbon-and-Optics/wle_p_analysis), released under the MIT License. Data and code are available at DOI: 10.5281/zenodo.19489452. Additional derived data sets and estimations are provided in the Supporting Information.

## Notes

Data and processing code repository; water column TP and SPM sample processing; metal and phosphorus extraction in sediments; SPM algorithm validation; additional statistical analyses for metals and phosphorus for sediment; and references (PDF)

## References

[1] Withers P. J. A., Jarvie H. P. (2008). Delivery and Cycling of Phosphorus in Rivers: A Review. Sci. Total Environ..

[2] Diaz R. J., Rosenberg R. (2008). Spreading
Dead Zones and Consequences for Marine Ecosystems. Science.

[3] Michalak A. M., Anderson E. J., Beletsky D., Boland S., Bosch N. S., Bridgeman T. B., Chaffin J. D., Cho K., Confesor R., Daloglu I., DePinto J. V., Evans M. A., Fahnenstiel G. L., He L., Ho J. C., Jenkins L., Johengen T. H., Kuo K. C., LaPorte E., Liu X., McWilliams M. R., Moore M. R., Posselt D. J., Richards R. P., Scavia D., Steiner A. L., Verhamme E., Wright D. M., Zagorski M. A. (2013). Record-Setting
Algal Bloom in Lake Erie Caused by Agricultural and Meteorological
Trends Consistent with Expected Future Conditions. Proc. Natl. Acad. Sci. U. S. A..

[4] Rucinski D. K., DePinto J. V., Beletsky D., Scavia D. (2016). Modeling Hypoxia
in
the Central Basin of Lake Erie under Potential Phosphorus Load Reduction
Scenarios. J. Great Lakes Res..

[5] Zhang H., Boegman L., Scavia D., Culver D. A. (2016). Spatial Distributions
of External and Internal Phosphorus Loads in Lake Erie and Their Impacts
on Phytoplankton and Water Quality. J. Great
Lakes Res..

[6] Cederwall J., Cott P. A. (2025). Rapidly Increasing Cyanobacteria Blooms in the Subarctic
Great Slave Lake: Observations from Indigenous, Local, and Scientific
Knowledge. Sci. Rep..

[7] Murphy R. R., Kemp W. M., Ball W. P. (2011). Long-Term Trends
in Chesapeake Bay
Seasonal Hypoxia, Stratification, and Nutrient Loading. Estuaries Coasts.

[8] Klump J. V., Brunner S. L., Grunert B. K., Kaster J. L., Weckerly K., Houghton E. M., Kennedy J. A., Valenta T. J. (2018). Evidence
of Persistent,
Recurring Summertime Hypoxia in Green Bay, Lake Michigan. J. Great Lakes Res..

[9] Obenour D. R., Michalak A. M., Zhou Y., Scavia D. (2012). Quantifying
the Impacts
of Stratification and Nutrient Loading on Hypoxia in the Northern
Gulf of Mexico. Environ. Sci. Technol..

[10] Steffen M. M., Davis T. W., Mckay R. M., Bullerjahn G. S., Krausfeldt L. E., Stough J. M. A., Neitzey M. L., Gilbert N. E., Boyer G. L., Johengen T. H., Gossiaux D. C., Burtner A. M., Palladino D., Rowe M., Dick G. J., Meyer K., Levy S., Boone B., Stumpf R., Wynne T., Zimba P. V., Gutierrez D. B., Wilhelm S. W. (2017). Ecophysiological
Examination of the Lake Erie Microcystis Bloom in 2014: Linkages between
Biology and the Water Supply Shutdown of Toledo, Ohio. Environ. Sci. Technol..

[11] Maccoux M. J., Dove A., Backus S. M., Dolan D. M. (2016). Total and Soluble
Reactive Phosphorus Loadings to Lake Erie: A Detailed Accounting by
Year, Basin, Country, and Tributary. J. Great
Lakes Res..

[12] Obenour D. R., Michalak A. M., Scavia D. (2015). Assessing Biophysical Controls on
Gulf of Mexico Hypoxia through Probabilistic Modeling. Ecological Applications.

[13] Ding S., Chen M., Gong M., Fan X., Qin B., Xu H., Gao S. S., Jin Z., Tsang D. C. W., Zhang C. (2018). Internal Phosphorus
Loading from Sediments Causes Seasonal Nitrogen Limitation for Harmful
Algal Blooms. Sci. Total Environ..

[14] Huang L., Fang H., He G., Jiang H., Wang C. (2016). Effects of
Internal Loading on Phosphorus Distribution in the Taihu Lake Driven
by Wind Waves and Lake Currents. Environ. Pollut..

[15] Puttonen I., Lukkari K., Miettunen E., Ropponen J., Tuomi L. (2024). Estimating
Internal Phosphorus Loading for a Water Quality Model Using Chemical
Characterisation of Sediment Phosphorus and Contrasting Oxygen Conditions. Sci. Total Environ..

[16] Orihel D. M., Schindler D. W., Ballard N. C., Graham M. D., O’Connell D. W., Wilson L. R., Vinebrooke R. D. (2015). The “Nutrient Pump:”
Iron-Poor Sediments Fuel Low Nitrogen-to-Phosphorus Ratios and Cyanobacterial
Blooms in Polymictic Lakes. Limnol. Oceanogr..

[17] Li J., Bai Y., Bear K., Joshi S., Jaisi D. (2017). Phosphorus Availability
and Turnover in the Chesapeake Bay: Insights from Nutrient Stoichiometry
and Phosphate Oxygen Isotope Ratios. J. Geophys.
Res. Biogeosci..

[18] Robertson D. M., Diebel M. W. (2020). Importance of Accurately Quantifying Internal Loading
in Developing Phosphorus Reduction Strategies for a Chain of Shallow
Lakes. Lake Reserv. Manag..

[19] Hanson P. C., Ladwig R., Buelo C., Albright E. A., Delany A. D., Carey C. C. (2023). Legacy Phosphorus
and Ecosystem Memory Control Future
Water Quality in a Eutrophic Lake. J. Geophys.
Res. Biogeosci..

[20] Kirol A. P., Morales-Williams A. M., Braun D. C., Marti C. L., Pierson O. E., Wagner K. J., Schroth A. W. (2024). Linking Sediment and Water Column
Phosphorus Dynamics to Oxygen, Temperature, and Aeration in Shallow
Eutrophic Lakes. Water Resour. Res..

[21] Sondergaard M., Kristensen P., Jeppesen E. (1992). Phosphorus Release
from Resuspended
Sediment in the Shallow and Wind-Exposed Lake Arreso, Denmark. Hydrobiologia.

[22] Huser B. J., Egemose S., Harper H., Hupfer M., Jensen H., Pilgrim K. M., Reitzel K., Rydin E., Futter M. (2016). Longevity
and Effectiveness of Aluminum Addition to Reduce Sediment Phosphorus
Release and Restore Lake Water Quality. Water
Res..

[23] Hipsey M. R., Bruce L. C., Boon C., Busch B., Carey C. C., Hamilton D. P., Hanson P. C., Read J. S., De Sousa E., Weber M., Winslow L. A. (2019). A General
Lake Model (GLM 3.0) for
Linking with High-Frequency Sensor Data from the Global Lake Ecological
Observatory Network (GLEON). Geosci. Model Dev..

[24] Matisoff G., Kaltenberg E. M., Steely R. L., Hummel S. K., Seo J., Gibbons K. J., Bridgeman T. B., Seo Y., Behbahani M., James W. F., Johnson L. T., Doan P., Dittrich M., Evans M. A., Chaffin J. D. (2016). Internal Loading of Phosphorus in
Western Lake Erie. J. Great Lakes Res..

[25] Verhamme E. M., Redder T. M., Schlea D. A., Grush J., Bratton J. F., DePinto J. V. (2016). Development of the
Western Lake Erie Ecosystem Model
(WLEEM): Application to Connect Phosphorus Loads to Cyanobacteria
Biomass. J. Great Lakes Res..

[26] Matisoff G., Carson M. L. (2014). Sediment Resuspension
in the Lake Erie Nearshore. J. Great Lakes Res..

[27] Baker D. B., Confesor R., Ewing D. E., Johnson L. T., Kramer J. W., Merryfield B. J. (2014). Phosphorus
Loading to Lake Erie from the Maumee, Sandusky
and Cuyahoga Rivers: The Importance of Bioavailability. J. Great Lakes Res..

[28] Muenich R. L., Kalcic M., Scavia D. (2016). Evaluating the Impact
of Legacy P
and Agricultural Conservation Practices on Nutrient Loads from the
Maumee River Watershed. Environ. Sci. Technol..

[29] Kemp W. M., Boynton W. R., Adolf J. E., Boesch D. F., Boicourt W. C., Brush G., Cornwell J. C., Fisher T. R., Glibert P. M., Hagy J. D., Harding L. W., Houde E. D., Kimmel D. G., Miller W. D., Newell R. I. E., Roman M. R., Smith E. M., Stevenson J. C. (2005). Eutrophication
of Chesapeake Bay: Historical Trends
and Ecological Interactions. Mar. Ecol.: Prog.
Ser..

[30] Ho J. C., Michalak A. M. (2017). Phytoplankton Blooms
in Lake Erie Impacted by Both
Long-Term and Springtime Phosphorus Loading. J. Great Lakes Res..

[31] Scavia D., DePinto J. V., Bertani I. (2016). A Multi-Model Approach
to Evaluating
Target Phosphorus Loads for Lake Erie. J. Great
Lakes Res..

[32] Kane D. D., Conroy J. D., Peter Richards R., Baker D. B., Culver D. A. (2014). Re-Eutrophication
of Lake Erie: Correlations between Tributary Nutrient Loads and Phytoplankton
Biomass. J. Great Lakes Res..

[33] Li H., Yang G., Ma J., Wei Y., Kang L., He Y., He Q. (2019). The Role of Turbulence
in Internal Phosphorus Release:
Turbulence Intensity Matters. Environ. Pollut..

[34] Sondergaard M., Jensen P. J., Jeppesen E. (2001). Retention
and Internal Loading of
Phosphorus in Shallow, Eutrophic Lakes. Scientific
World.

[35] Tammeorg O., Nürnberg G. K., Tõnno I., Kisand A., Tuvikene L., Nõges T., Nõges P. (2022). Sediment Phosphorus Mobility in Võrtsjärv,
a Large Shallow Lake: Insights from Phosphorus Sorption Experiments
and Long-Term Monitoring. Sci. Total Environ..

[36] Conroy J. D., Kane D. D., Dolan D. M., Edwards W. J., Charlton M. N., Culver D. A. (2005). Temporal Trends
in Lake Erie Plankton Biomass: Roles
of External Phosphorus Loading and Dreissenid Mussels. J. Great Lakes Res..

[37] Søndergaard M., Jeppesen E., Lauridsen T. L., Skov C., Van Nes E. H., Roijackers R., Lammens E., Portielje R. (2007). Lake Restoration:
Successes, Failures and Long-Term Effects. J.
Appl. Ecol..

[38] Jeppesen E., Søndergaard M., Jensen J. P., Havens K. E., Anneville O., Carvalho L., Coveney M. F., Deneke R., Dokulil M. T., Foy B., Gerdeaux D., Hampton S. E., Hilt S., Kangur K., Köhler J., Lammens E. H. H. R., Lauridsen T. L., Manca M., Miracle M. R., Moss B., Nõges P., Persson G., Phillips G., Portielje R., Romo S., Schelske C. L., Straile D., Tatrai I., Willén E., Winder M. (2005). Lake Responses to Reduced Nutrient
Loading - An Analysis of Contemporary Long-Term Data from 35 Case
Studies. Freshw. Biol..

[39] Nechad B., Ruddick K. G., Park Y. (2010). Calibration
and Validation of a Generic
Multisensor Algorithm for Mapping of Total Suspended Matter in Turbid
Waters. Remote Sens. Environ..

[40] Yuan F., Li H., Kakarla R., Kasden C., Yao S., Xue B., Sun Y. (2020). Variability
of Sedimentary Phosphorus Fractions in the Western and
Sandusky Basins of Lake Erie. J. Great Lakes
Res..

[41] Wu T., Qin B., Brookes J. D., Yan W., Ji X., Feng J. (2019). Spatial Distribution
of Sediment Nitrogen and Phosphorus in Lake Taihu from a Hydrodynamics-Induced
Transport Perspective. Sci. Total Environ..

[42] Hansen P. S., Phlips E. J., Aldridge F. J. (1997). The Effects
of Sediment Resuspension
on Phosphorus Available for Algal Growth in a Shallow Subtropical
Lake, Lake Okeechobee. Lake Reserv. Manag..

[43] Jabbari A., Ackerman J. D., Boegman L., Zhao Y. (2021). Increases in Great
Lake Winds and Extreme Events Facilitate Interbasin Coupling and Reduce
Water Quality in Lake Erie. Sci. Rep..

[44] Michalak A. M. (2016). Study Role
of Climate Change in Extreme Threats to Water Quality. Nature.

[45] Annex 4 Adaptive Management Task Team . Binational Adaptive Management Evaluation for Lake Erie (2017–2021); International Joint Commission: Windsor, Ontario, 2024.

[46] Jarvie H. P., Johnson L. T., Sharpley A. N., Smith D. R., Baker D. B., Bruulsema T. W., Confesor R. (2017). Increased Soluble Phosphorus Loads
to Lake Erie: Unintended Consequences of Conservation Practices?. J. Environ. Qual..

[47] U.S. Environmental Protection Agency Method 365.1, Revision 2.0: Determination of Phosphorus by Semi-automated Colorimetry; U.S EPA: Cincinnati, OH, 1993.

[48] American Public Health Association (APHA); American Water Works Association (AWWA) ; Water Environment Federation (WEF). 2540 Solids. In Standard Methods for the Examination of Water and Wastewater, 21st ed.; APHA: Washington, DC, 2005; pp 55–60.

[49] Boss E., Taylor L., Gilbert S., Gundersen K., Hawley N., Janzen C., Johengen T., Purcell H., Robertson C., Schar D. W. H., Smith G. J., Tamburri M. N. (2009). Comparison
of Inherent Optical Properties as a Surrogate for Particulate Matter
Concentration in Coastal Waters. Limnol. Oceanogr.
Methods.

[50] Woźniak S. B., Meler J., Lednicka B., Zdun A., Stoń-Egiert J. (2011). Inherent Optical
Properties of Suspended Particulate Matter in the Southern Baltic
Sea. Oceanologia.

[51] U.S. Environmental Protection Agency Method 3050B: Acid Digestion of Sediment, Sludges and Soils, Revision 2; U.S EPA: Washington, DC, 1996.

[52] Yuan F., Chaffin J. D., Xue B., Wattrus N., Zhu Y., Sun Y. (2018). Contrasting Sources
and Mobility of Trace Metals in Recent Sediments
of Western Lake Erie. J. Great Lakes Res..

[53] Jensen H. S., Thamdrup B. (1993). Iron-Bound Phosphorus
in Marine Sediments as Measured
by Bicarbonate-Dithionite Extraction. Hydrobiologia.

[54] Paytan A., Roberts K., Watson S., Peek S., Chuang P. C., Defforey D., Kendall C. (2017). Internal Loading of Phosphate in
Lake Erie Central Basin. Sci. Total Environ..

[55] SEAL Analytical EPA-118-A Rev. 5: Determination of Ortho-Phosphate–P in Drinking, Saline, and Surface Waters, and Domestic and Industrial Wastes; SEAL Analytical: Mequon, WI, USA, 2012.

[56] SEAL Analytical . EPA-134-A Rev. 5: Phosphorus–P, Total. In Surface and Saline Waters, and Domestic and Industrial Wastes; SEAL Analytical: Mequon, 2013; Vol. WI.

[57] Fitzgerald S. A., Klump J. V., Swarzenski P. W., Mackenzie R. A., Richards K. D. (2001). Beryllium-7 as a Tracer of Short-Term
Sediment Deposition
and Resuspension in the Fox River Wisconsin. Environ. Sci. Technol..

[58] Larsen I. L., Cutshall N. H. (1981). Direct Determination of 7Be in Sediments. Earth Planet. Sci. Lett..

[59] Sentinel-3 Ocean and Land Colour Instrument (OLCI) Level-0 and Level-1 Data . Level-1 and Atmosphere Archive and Distribution System (LAADS) Distributed Active Archive Center (DAAC); NASA Goddard Space Flight Center, 2025.

[60] Steinmetz F., Deschamps P.-Y., Ramon D. (2011). Atmospheric Correction in Presence
of Sun Glint: Application to MERIS. Opt. Express.

[61] Mobley C. D. (1999). Estimation
of the Remote-Sensing Reflectance from Above-Surface Measurements. Appl. Opt..

[62] Zibordi, G. ; Voss, K. J. ; Johnson, B. C. ; Mueller, J. L. IOCCG Ocean Optics and Biogeochemistry Protocols for Satellite Ocean Colour Sensor Validation; IOCCG: Dartmouth, NS, Canada, 2019; Vol. 3.0. 10.25607/OBP-691

[63] Groetsch P. M. M., Gege P., Simis S. G. H., Eleveld M. A., Peters S. W. M. (2017). Validation
of a Spectral Correction Procedure for Sun and Sky Reflections in
Above-Water Reflectance Measurements. Opt. Express.

[64] Green M. A., Aller R. C., Cochran J. K., Lee C., Aller J. Y. (2002). Bioturbation
in Shelf/Slope Sediments off Cape Hatteras, North Carolina: The Use
of 234Th, Chl-a, and Br- to Evaluate Rates of Particle and Solute
Transport. Stud. Oceanogr..

[65] Nittrouer C. A., Demaster D. J., Mckee B. A., Cutshall N. H., Larsen I. L. (1984). The Effect
of Sediment Mixing on Pb-210 Accumulation Rates for the Washington
Continental Shelf. Mar. Geol..

[66] Klump J. V., Edgington D. N., Sager P. E., Robertson D. M. (1997). Sedimentary
Phosphorus Cycling and a Phosphorus Mass Balance for the Green Bay
(Lake Michigan) Ecosystem. Can. J. Fish. Aquat.
Sci..

[67] Niu Q., Xia M., Ludsin S. A., Chu P. Y., Mason D. M., Rutherford E. S. (2018). High-Turbidity
Events in Western Lake Erie during Ice-Free Cycles: Contributions
of River-Loaded vs. Resuspended Sediments. Limnol.
Oceanogr..

[68] Bathymetry of Lake Erie and Lake Saint Clair (Bathymetric Contour Shapefiles) . National Geophysical Data Center; NOAA National Centers for Environmental Information: Silver Spring, MD, 1999. 10.7289/V5KS6PHK.

[69] Jensen H. S., Kristensen P., Jeppesen E., Skytthe A. (1992). Iron:Phosphorus Ratio
in Surface Sediment as an Indicator of Phosphate Release from Aerobic
Sediments in Shallow Lakes. Hydrobiologia.

[70] Station THRO1 – Toledo, OH . National Data Buoy Center; NOAA.

[71] Wang Y. T., Zhang T. Q., Zhao Y. C., Ciborowski J. H. H., Zhao Y. M., O’Halloran I.
P., Qi Z. M., Tan C. S. (2021). Characterization of Sedimentary Phosphorus in Lake
Erie and On-Site Quantification of Internal Phosphorus Loading. Water Res..

[72] Seo, J. Sediment Mass and Nutrient Accumulation Rates in Lake Erie Using Geographic Information System. Discussions 2015, 12(1)15 22.

[73] Kemp A. L. W., MacInnis G. A., Harper N. S. (1977). Sedimentation
Rates and a Revised
Sediment Budget for Lake Erie. J. Great Lakes
Res..

[74] Matisoff G., Wilson C. G., Whiting P. J. (2005). The 7Be/210PbXS
Ratio as an Indicator
of Suspended Sediment Age or Fraction New Sediment in Suspension. Earth Surf. Process. Landf..

[75] Sayers M. J., Grimm A. G., Shuchman R. A., Bosse K. R., Fahnenstiel G. L., Ruberg S. A., Leshkevich G. A. (2019). Satellite Monitoring of Harmful Algal
Blooms in the Western Basin of Lake Erie: A 20-Year Time-Series. J. Great Lakes Res..

[76] Missimer T. M., Thomas S., Rosen B. H. (2021). Legacy Phosphorus
in Lake Okeechobee
(Florida, USA) Sediments: A Review and New Perspective. Water.

[77] Albright E. A., Wilkinson G. M. (2022). Sediment
Phosphorus Composition Controls Hot Spots
and Hot Moments of Internal Loading in a Temperate Reservoir. Ecosphere.

[78] Tammeorg O., Nürnberg G. K., Tõnno I., Toom L., Nõges P. (2024). Spatio-Temporal
Variations in Sediment Phosphorus Dynamics in a Large Shallow Lake:
Mechanisms and Impacts of Redox-Related Internal Phosphorus Loading. Sci. Total Environ..

[79] Bocaniov S. A., Scavia D., Van Cappellen P. (2023). Long-Term
Phosphorus Mass-Balance
of Lake Erie (Canada-USA) Reveals a Major Contribution of in-Lake
Phosphorus Loading. Ecol. Inform..

