# Supplementary material for: Sediment Resuspension as a System-Wide Driver of Legacy and Bioavailable Phosphorus Release in Lake Erie
Source: Environ Sci Technol. 2026 Apr 10;60(16):12207–18. doi: 10.1021/acs.est.5c17601 (PMC13130958; doi:10.1021/acs.est.5c17601)
Supplement: Supplementary file 1 [file es5c17601_si_001.pdf]

Supporting Information (SI) for

Sediment resuspension as a system-wide driver of legacy and bioavailable phosphorus release in Lake Erie

Anshula Dhiman[a], Trevor Holm[a], Kendra Herweck[a], Audrey Ciochetto[a,b], Timothy Wahl[c], Fasong Yuan[a], J. Val Klump[c], Brice K. Grunert[a]*

[a]Biological Geological and Environmental Sciences, Cleveland State University, Cleveland, OH 44115, USA

[b]Graduate School of Oceanography, University of Rhode Island, Narragansett, RI 02882, USA

[c]School of Freshwater Sciences, University of Wisconsin-Milwaukee, Milwaukee, WI 53204, USA

***Corresponding author:** a.dhiman25@vikes.csuohio.edu

Supporting Information content

1. Data and Processing Code Repository (Pages S4 – S6)

1.1 Content of the repository S4

1.2 Repository Structure (from README) S5

1.3 Reproducibility steps S6

2. 3 SI Methods (Pages S7 – S8)

2.1 Water column TP and SPM sample processing S7

2.2 Metal and phosphorus extraction in sediments S7

2.3 SPM algorithm validation S8

3. 5 SI Figures (Pages S9 – S18)

Figure S1. Validation of modeled versus in situ SPM across Great Lakes sites. S9

Figure S2. Measured and modeled ^{7}Be activity vertical profiles S11

Figure S3. Mixing rates (D_b) based on ^{7}Be profiles S13

Figure S4. Temporal change of measured ^{7}Be inventories and reference decay curves. S15

Figure S5. Linear fit of chlorophyll and total phosphorus (TP) versus suspended particulate matter (SPM) S17

4. 5 SI Tables (Pages S18 – S24)

Table S1. Measured SPM activities of ^{7}Be in western Lake Erie during 2023. .... S18

Table S2: Calculated water column residence times and settling rates. S19

Table S3: Measured A_T (Surface ^{7}Be activity: 0–1cm) and penetration depths, and calculated sediment mixing coefficient (D_b) and mixing depths (Z_{mix} and h). S20

Table S4: One-way ANOVA results showing variations of P fraction concentrations across depths (0–5 cm). S22

Table S5: One-way ANOVA results showing variations of metal concentrations across years: 2016, 2022, and 2023... S23

5. 14 SI References (Pages S24 – S26)

1. Data and Processing Code Repository

All the code and related data used in the analysis of this study are available in the public repository 'wle_p_analysis' (https://github.com/Carbon-and-Optics/wle_p_analysis),[1] released under the MIT license.

1.1 Content of the repository

The repository contains the analysis data, scripts for sediment phosphorus, Beryllium-7 (^7Be), satellite-based suspended particulate matter (SPM) retrieval algorithms, and estimation of basin-wide bioavailable phosphorus (P) release from satellite-derived SPM observations. Specifically, the repository provides:

Python scripts for processing the Sentinel-3 satellite observations, retrieving SPM, and generating ΔSPM maps used in system-scale resuspension analysis.

Python scripts for calculating sediment mixing parameters from ^7Be profiles, resuspension-derived bioavailable P release, and basin-wide internal loading estimates.

Data folders containing original environmental datasets (bathymetry, shapefiles, remote sensing inputs), processed datasets, and intermediate products used to generate figures and tables in the manuscript and SI.

1.2 Repository Structure (from README)

The GitHub repository includes a README file detailing dependencies, programming language versions (Python 3.10), required libraries, and a step-by-step guide to rerun all the data processing, analyses, and figure generation. All the required datasets required for analysis (no external downloads needed):

sentinel_data/: Level 1B Top-Of-Atmosphere (TOA) radiance product from the European Space Agency's Sentinel-3A Ocean and Land Color Instrument (OLCI) from NASA's Atmosphere Archive and Distribution System Distributed Active Archive Center (LAADS.DAAC),[2] which is POLYMER corrected for May 18 and 26, 2023, including derived SPM outputs using SPM algorithm[3] used for system-scale resuspension mapping.

lake_erie_shapefiles/: Shapefiles representing Lake Erie basin boundaries from NOAA National Centers for Environmental Information (NCEI)[4] contours, and shorelines used for spatial masking and domain definition.

sediment_data/: Sediment core data including ^{7}Be activities, P-fractionation (total phosphorus (TP), Bioavailable-P), porosity, bulk density, and metal concentrations.

water_data/: In situ water quality measurements (SPM, TP, soluble reactive phosphorus (SRP), Chlorophyll-a) used to validate SPM-derived remote-sensing products.

THRO1_WIND_DATA/: Meteorological wind time-series files from NOAA[5] National Data Buoy Center (NDBC), Station THRO1 used to characterize resuspension-forcing conditions.

GSHHS/: High-resolution coastline polygons provided by NOAA NCEI[6] used for accurate masking and map construction.

Configuration file (config.yml) specifying directory paths and user settings. requirements.txt provides list of required Python dependencies needed to reproduce the analysis environment.

1.3 Reproducibility steps

1. Clone the repository: git clone https://github.com/Carbon-and-Optics/wle_p_analysis

2. Install the required Python libraries using: pip install -r requirements.txt.

3. Open the notebook: analysis/manuscript_and_SI_figures_and_analysis.ipynb

4. Execute all cells to reproduce figures, tables, calculations and statistical analysis present in manuscript and SI.

2. SI Methods

2.1 Water column TP and SPM sample processing

Water column TP, which includes both particulate and dissolved P, was determined by persulfate digestion following EPA Method 365.1.[7] A 50 mL aliquot of each sample was digested with 1 mL of 5.5 M H_2SO_4 and 0.4 g of $(NH_4)_2S_2O_8$ (ammonium persulfate). The persulfate oxidation converts all phosphorus forms, including organic phosphorus compounds to orthophosphate.

The collected SPM filters were dried at 105 °C for 24 hours and reweighed to determine SPM mass as the difference between the initial and final filter weights.[8–10] SPM concentration was then calculated by dividing the SPM mass by the volume of water filtered.

2.2 Metal and phosphorus extraction in sediments

Metals were extracted following USEPA Method 3050B.[11] Dried, homogenized sediment (0.2 g) was treated with 10 mL of 1:1 HNO_3 and heated to 95 ± 5 °C for 10–15 minutes. Concentrated HNO_3 (5 mL) was then added in successive aliquots with continued heating until the cessation of brown fumes confirmed complete sample oxidation. The volume was reduced to ~5 mL before adding water (2 mL) and 30% H_2O_2 to further oxidize organic matter. Following a second volume reduction to ~5 mL, concentrated HCl (10 mL) was added, and the mixture was refluxed at 95 ± 5 °C for 15 minutes. The cooled digestate was diluted to 100 mL with deionized water, filtered, and analyzed by ICP-MS.

Fractionation followed a modified five-step sequential extraction[12,13] of 0.25 g of sediment in 100 mL centrifuge tubes using: (1) 0.46 M NaCl for loosely-bound P, (2) sodium bicarbonate-dithionite (NaBD) for redox-sensitive P (with air bubbling to remove residual dithionite), (3) 0.1 M NaOH for Al/Fe oxide-bound P (overnight shaking), and (4) 0.5 M HCl for Ca-bound P. Each step included a 15-minute rinse and centrifugation at 4,000 rpm. Supernatants from each step were preserved with 4.5 M H_2SO_4 (except HCl extract) and filtered, with NaOH extracts acidified after filtration. Residual organic P was extracted from the sediment residue by boiling with 50 mL Milli-Q water, 1 mL of 5.5 M H_2SO_4, and 0.4 g of $(NH_4)_2S_2O_8$ (EPA Method 365.1). After boiling to ~10 mL, the solution was cooled, diluted to 50 mL with Milli-Q water, and filtered.

2.3 SPM algorithm validation

We evaluated the performance of the Nechad et al.[3] single-band SPM algorithm using 117 in situ water column measurements for SPM collected concurrently with surface radiometric data from a Spectra Vista Corporation (SVC spectroradiometer) at the same sampling sites across the Great Lakes in 2023 and 2024 (Figure S1). The reflectance data were corrected using the 3C method[14] before comparison with in situ SPM observations, as described in the Methods section of the main manuscript.

The results from applying the equations (Equations 1–7) explained in Material and methods section from main manuscript are shown in Supplementary Figures S2, S3, S4 and Table S1, S2, and S3.

3. SI Figures

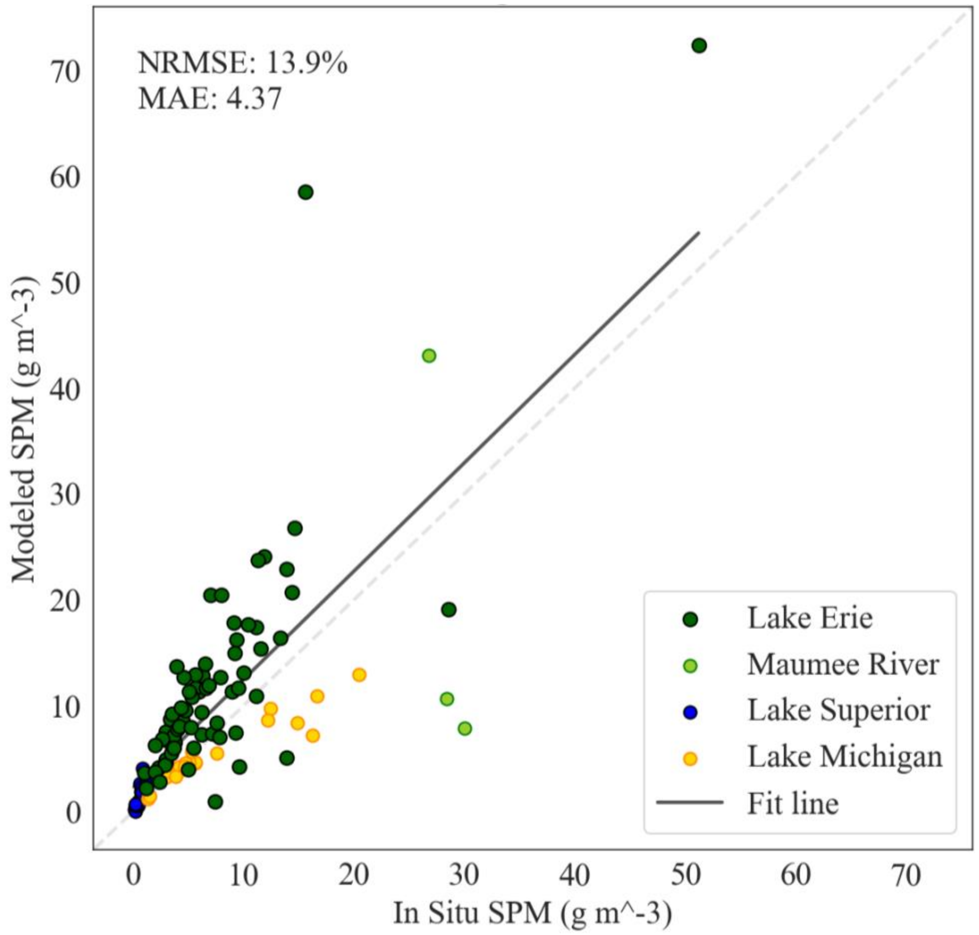

Figure S1. Validation of modeled versus in situ SPM across Great Lakes sites. Comparison of in situ measured SPM and Nechad et al.[3] estimated SPM using in situ reflectance ($R_{rs}(\lambda)$, n = 117). The solid black fit line shows the least-squares regression fit ($R^2 = 0.44$), while the dashed gray line indicates the 1:1 reference.

(a)

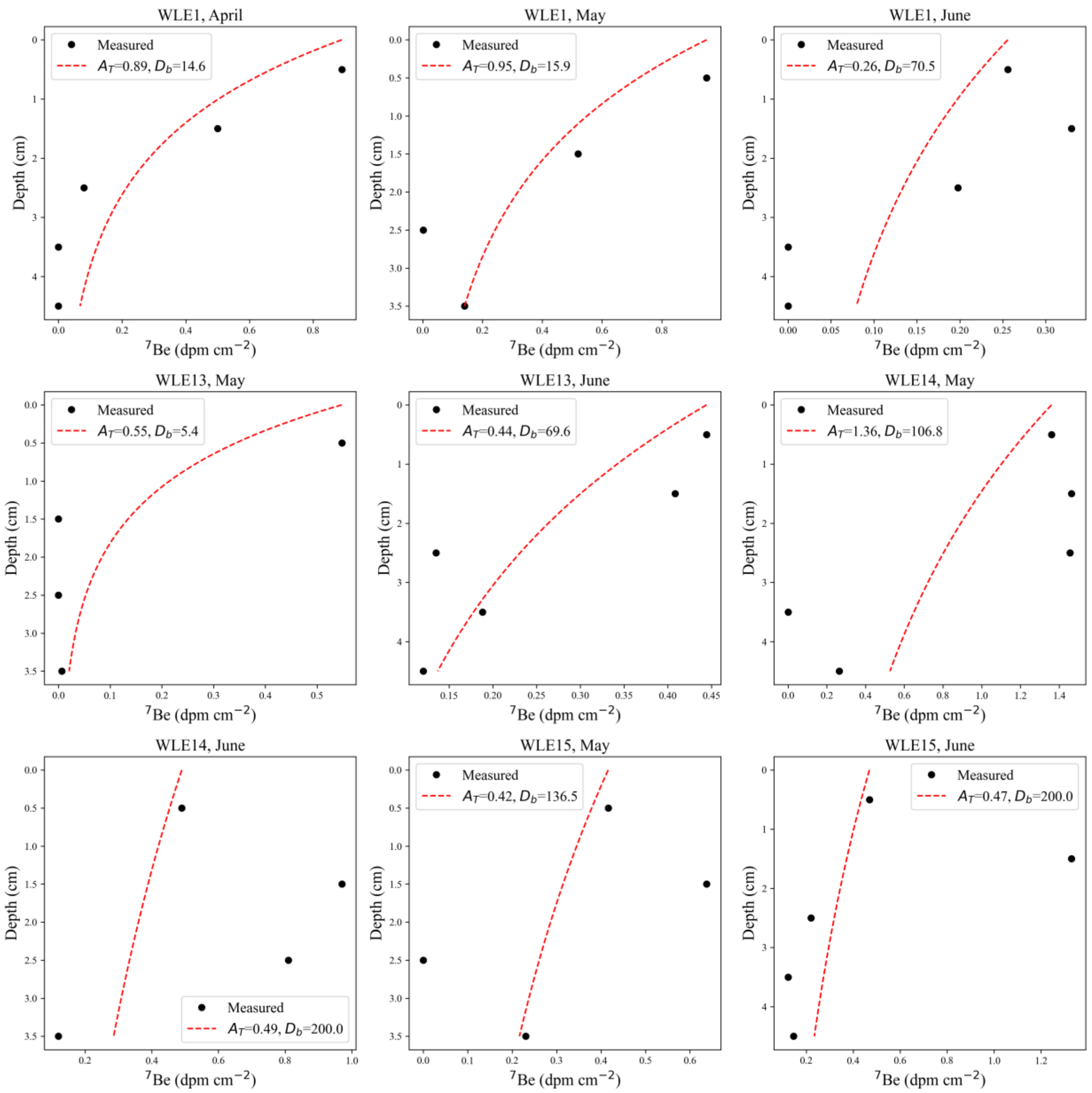
WLE1, April
Measured
A_T=0.89, D_b=14.6
WLE1, May
Measured
A_T=0.95, D_b=15.9
WLE1, June
Measured
A_T=0.26, D_b=70.5
WLE13, May
Measured
A_T=0.55, D_b=5.4
WLE13, June
Measured
A_T=0.44, D_b=69.6
WLE14, May
Measured
A_T=1.36, D_b=106.8
WLE14, June
Measured
A_T=0.49, D_b=200.0
WLE15, May
Measured
A_T=0.42, D_b=136.5
WLE15, June
Measured
A_T=0.47, D_b=200.0
Depth (cm)
^{7}Be (dpm cm^{-2})

(b)

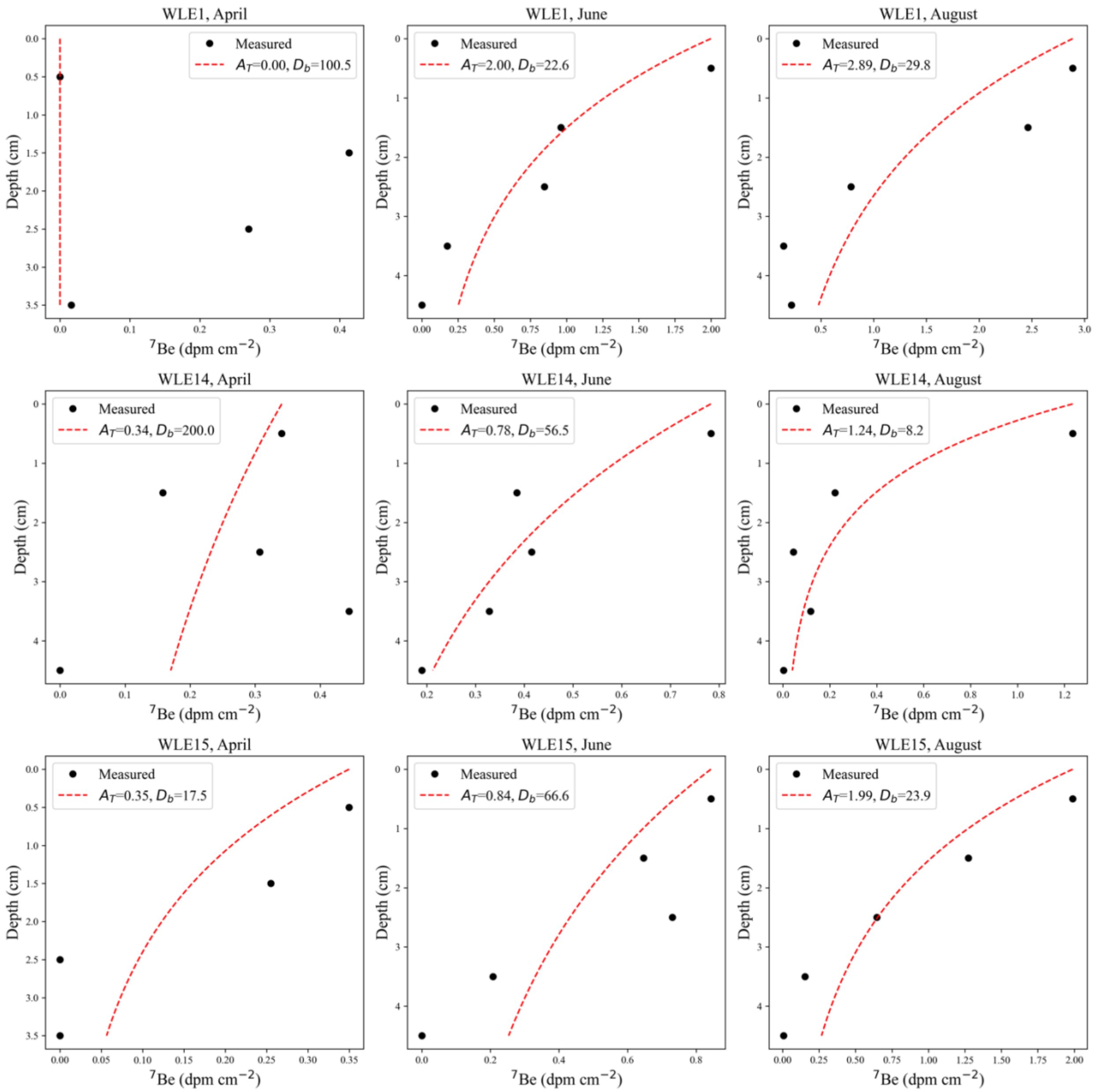

Figure S2. Measured and modeled ^{7}Be activity vertical profiles. Vertical profiles of measured and modeled ^{7}Be activity (using Equation 1) in surface sediment at western Lake

Erie stations during (a) 2023 and (b) 2024. Filled black symbols indicate in situ measured ^{7}Be activities (dpm cm^{-3}), and dashed curves show exponential model fits using surface (0–1) activity (A_T) and best-fit apparent mixing coefficient (D_b, cm^2 year^{-1}). Each panel represents an individual station and sampling month, illustrating seasonal and interannual variation in sediment mixing and ^{7}Be penetration depth.

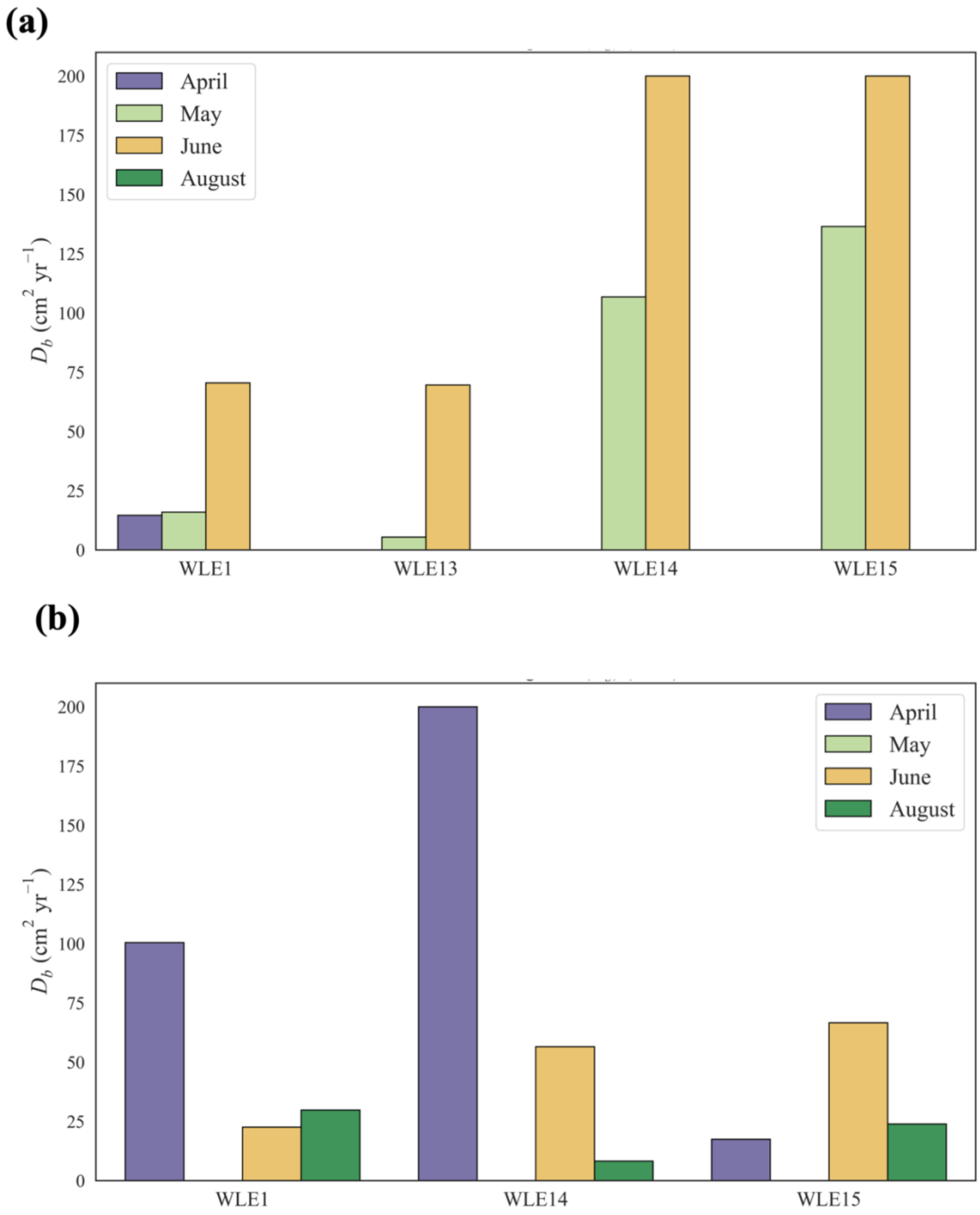

Figure S3. Mixing rates (D_b) based on ^{7}Be profiles. Seasonal variations of sediment-mixing coefficients (D_b) at coring sites in western Lake Erie in (a) 2023 and (b) 2024.

Mixing rate (cm^2 yr^{-1}) was derived from ^7Be profiles for each monthly core as shown in Figure S2.

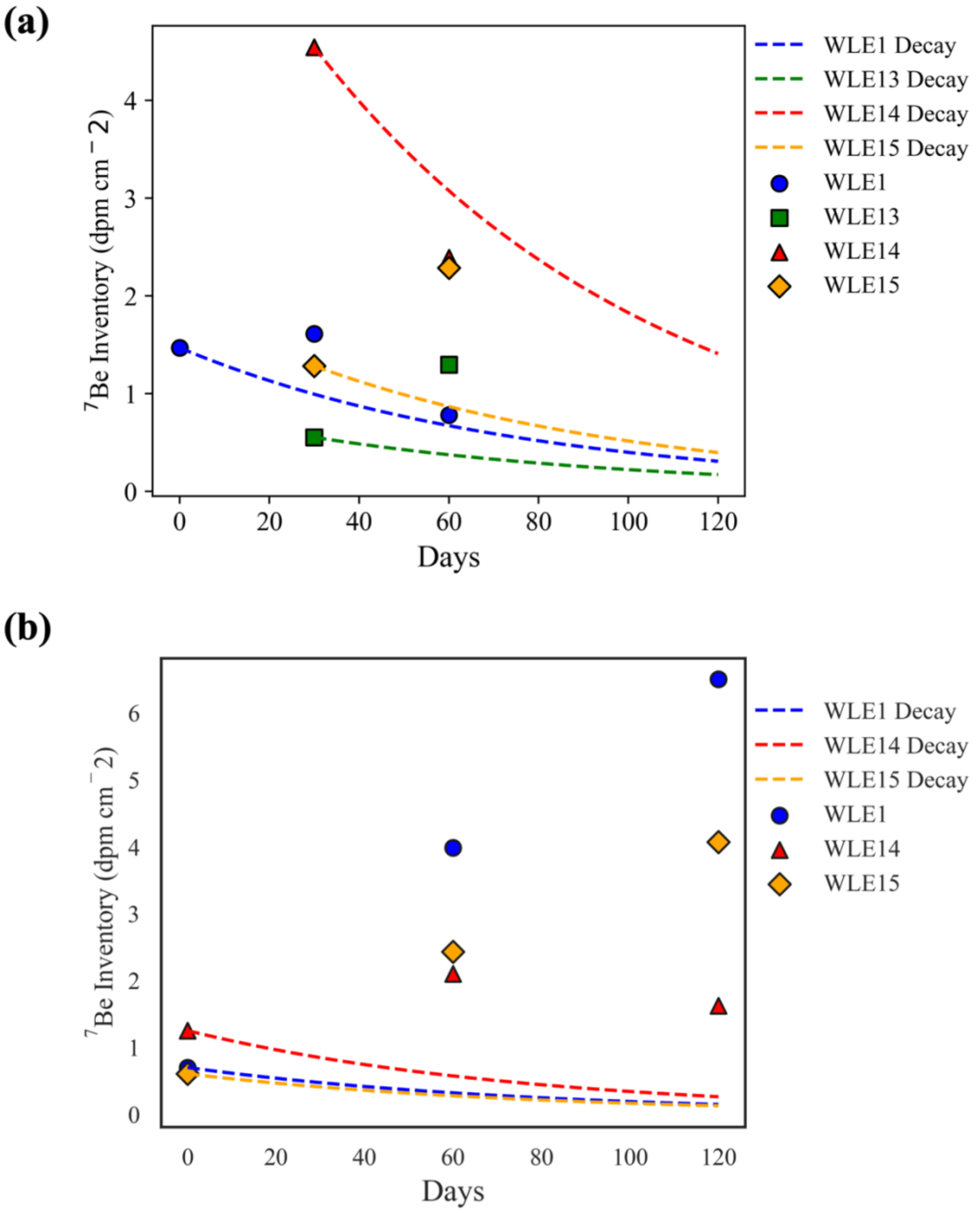

Figure S4. Temporal change of measured ^{7}Be inventories and reference decay curves.

^{7}Be total inventories and natural-decay reference curves across (a) 2023 and (b) 2024, with different markers showing core-integrated ^{7}Be inventories (dpm cm^{-2}) at WLE1, WLE13,

WLE14, and WLE15 by sampling month and the x-axis is days since April, representing day 0, May as day 30, June as day 60 and August as day 120. Dashed curves are the expected exponential decay of each station's earliest measured inventory (half-life = 53.3 days). Deviations above the curve indicate net addition deposition, whereas values below indicate loss (resuspension).

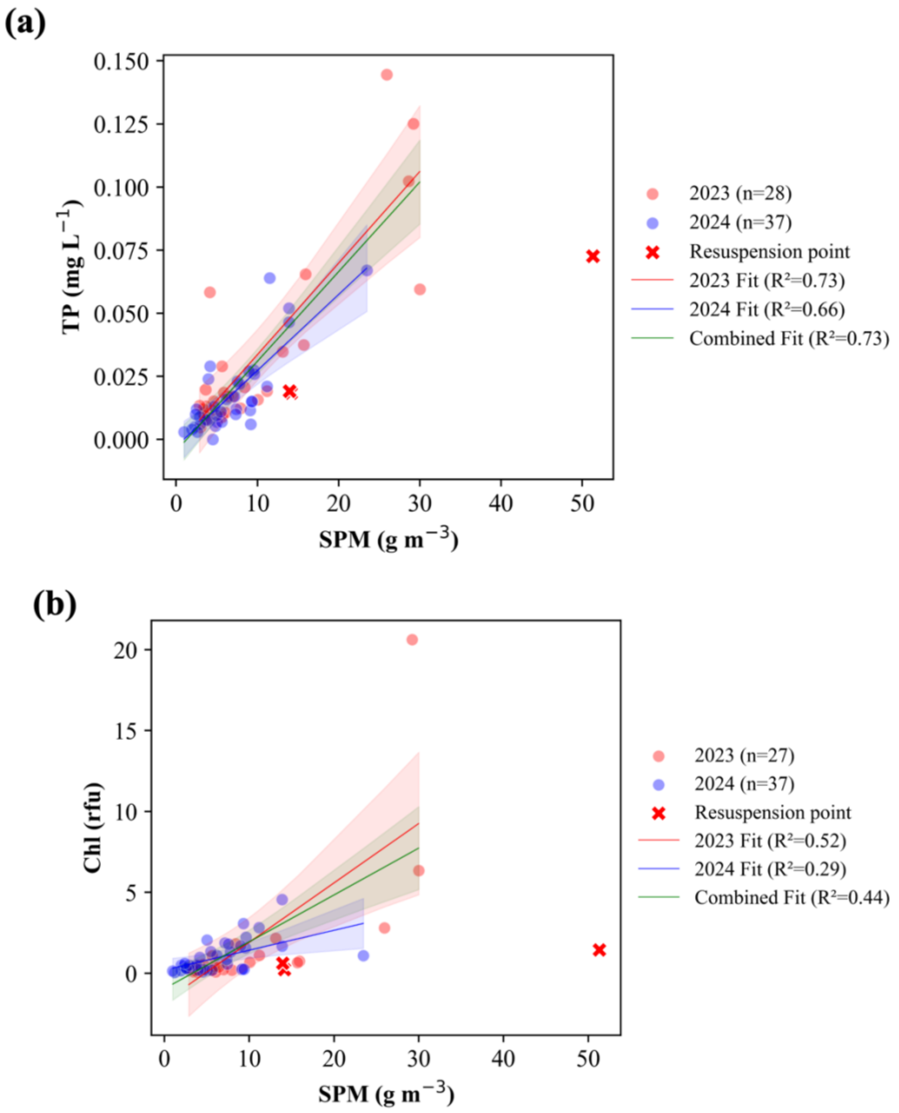

Figure S5. Linear fit of chlorophyll and total phosphorus (TP) versus suspended particulate matter (SPM). Relationships between (a) TP and (b) Chlorophyll (rfu) and SPM in 2023 (blue) and 2024 (red). Linear fits are with shaded 95% confidence intervals. Combined fit includes all 2023 and 2024 data excluding the resuspension data in western Lake Erie.

4. SI Tables

Table S1. Measured SPM activities of ^{7}Be in western Lake Erie during 2023.

Station	Month	^{7}Be (dpm L^{-1})	^{7}Be (dpm g^{-1})
WLE16	May	0.038	2.72
WLE13	May	0.3394	6.62
WLE14	May	0.1383	39.89
WLE15	May	0.089	26.57
WLE1	May	0.208	18.58
WLE1	June	0.2145	7.15
WLE15	June	0.0635	6.31

Table S2: Calculated water column residence times and settling rates.

Station	Residence Time (T_{res}) (days)	Settling Rates (m $days^{-1}$)
WLE1 (05/23/23)	2.2	2.3
WLE14 (05/23/23)	2	4.3
WLE15 (05/23/23)	5.6	1.9
WLE1 (06/21/23)	10.6	0.5
WLE15 (06/21/23)	2.3	4.7

Table S3: Measured A_T (Surface ^{7}Be activity: 0–1cm) and penetration depths, and calculated sediment mixing coefficient (D_b) and mixing depths (Z_{mix} and h).

**Station**	**Month**	**A_T (dpm cm^{-3})**	**D_b (cm^2 yr^{-1})**	**Observed penetration depth (cm)**	**Mixing depth (Z_{mix})**	**Mixing depth (h)**
**2023**						
WLE1	April	0.89	14.60	3	4.4	2.6
WLE1	May	0.95	15.94	4	3.4	2.5
WLE1	June	0.26	70.51	3	1.3	7.3
WLE13	May	0.55	5.43	4	4.7	1.5
WLE13	June	0.44	69.6	5	5	5.4
WLE14	May	1.36	106.76	5	7.8	6.7
WLE14	June	0.49	200	4	9.1	9.2
WLE15	May	0.42	136.51	4	2.3	5.5
WLE15	June	0.47	200	5	7.6	9.2
**2024**						
WLE1	April	0	100.5	4	14.9	6.5
WLE1	June	1.9989	22.56	4	5.3	3.1
WLE1	August	2.8874	29.8	5	6.4	3.5
WLE14	April	0.3409	200	4	1.7	9.2

WLE14	June	0.7833	56.48	5	4.9	4.9
WLE14	August	1.2354	8.25	5	3.1	1.9
WLE15	April	0.3497	16.23	2	0.6	2.6
WLE15	June	0.8431	66.61	4	5.2	5.3
WLE15	August	1.9893	23.89	5	5.7	3.2

Table S4: One-way ANOVA results showing variations of P fraction concentrations across depths (0–5 cm).

**Fraction**	**F-statistic**	**p-value**
Loosely-bound	0.577	0.681
Redox-sensitive	0.339	0.850
Al&Fe oxide-bound	0.230	0.920
Ca-bound	0.187	0.944
Organic P	0.610	0.494
TP	0.610	0.658

Statistical analysis used $n = 45$ observations, with 4 degrees of freedom between depths and 40 degrees of freedom within groups. No depth-dependent differences were statistically significant (at $p < 0.05$).

Table S5: One-way ANOVA results showing variations of metal concentrations across years: 2016, 2022, and 2023.

Metal	F-value	p-value
Sr	0.671	0.647
Mo	6.804	0.000
Cd	1.009	0.422
Pb	1.506	0.205
Cr	0. 856	0.517
Mn	7.835	0.000
Fe	0.875	0.504
Co	3.261	0.013
Ni	1.455	0.221
Cu	1.152	0.346
Zn	1.88	0.115

Statistical analysis used $n = 56$ observations, with 5 degrees of freedom among years (reflecting seasonal data for 2022 and 2023) and 50 degrees of freedom within groups. Significant differences were detected for Mo, Co and Mn ($p < 0.05$).

5. SI References

(1) Anshula Dhiman, K. H. T. H. Wle_p_analysis: Western Lake Erie Phosphorus Analysis. GitHub 2025.

(2) Sentinel-3 Ocean and Land Colour Instrument (OLCI) Level-0 and Level-1 Data. *Level-1 and Atmosphere Archive and Distribution System (LAADS) Distributed Active Archive Center (DAAC)*; NASA Goddard Space Flight Center, 2025. https://ladsweb.modaps.eosdis.nasa.gov/missions-and-measurements/science-domain/olci-L0L1/ (accessed 2025-10-29).

(3) Nechad, B.; Ruddick, K. G.; Park, Y. Calibration and Validation of a Generic Multisensor Algorithm for Mapping of Total Suspended Matter in Turbid Waters. *Remote Sens. Environ.* **2010**, *114* (4), 854–866, DOI: 10.1016/j.rse.2009.11.022

(4) Bathymetry of Lake Erie and Lake Saint Clair (Bathymetric Contour Shapefiles). *National Geophysical Data Center*; NOAA National Centers for Environmental Information: Silver Spring, MD, 1999. https://www.ngdc.noaa.gov/mgg/greatlakes/erie.html (accessed 2025-10-29).

(5) Station THRO1 – Toledo, OH. *National Data Buoy Center*; NOAA. *https://www.ndbc.noaa.gov/station_page.php?station=thro1 (accessed 2025-10-30).*

(6) Wessel, P. ; S. W. H. F. GSHHG: Global Self-Consistent, Hierarchical, High-Resolution Geography Database. NOAA National Centers for Environmental Information (NCEI): Boulder, CO 2019.

(7) U.S. Environmental Protection Agency. *Method 365.1, Revision 2.0: Determination of Phosphorus by Semi-Automated Colorimetry*; U.S EPA: Cincinnati, OH, 1993.

(8) American Public Health Association (APHA); American Water Works Association (AWWA); Water Environment Federation (WEF). 2540 Solids. In *Standard Methods for the Examination of Water and Wastewater,* 21st ed.; APHA: Washington, DC*,* 2005; pp 55–60.

(9) Boss, E.; Taylor, L.; Gilbert, S.; Gundersen, K.; Hawley, N.; Janzen, C.; Johengen, T.; Purcell, H.; Robertson, C.; Schar, D. W. H.; Smith, G. J.; Tamburri, M. N. Comparison of Inherent Optical Properties as a Surrogate for Particulate Matter Concentration in Coastal Waters. *Limnol. Oceanogr. Methods* **2009**, *7* (11), 803–810, DOI: 10.4319/lom.2009.7.803

(10) Woźniak, S. B.; Meler, J.; Lednicka, B.; Zdun, A.; Stoń-Egiert, J. Inherent Optical Properties of Suspended Particulate Matter in the Southern Baltic Sea. *Oceanologia* **2011**, *53* (3), 691–729, DOI: 10.5697/oc.53-3.691.

(11) USEPA. *Method 3050B: Acid Digestion of Sediment, Sludges and Soils, Revision 2*; Washington, DC, 1996.

(12) Jensen, H S.; Thamdrup, B. Iron-Bound Phosphorus in Marine Sediments as Measured by Bicarbonate-Dithionite Extraction. *Hydrobiologia* **1993**, *253*, 47–59, DOI: 10.1007/BF00050721

(13) Lukkari, K.; Hartikainen, H.; Leivuori, M. Fractionation of Sediment Phosphorus Revisited. I: Fractionation Steps and Their Biogeochemical Basis. *Limnol. Oceanogr. Methods* **2007**, *5* (12), 433–444, DOI: 10.4319/lom.2007.5.433

(14) Groetsch, P. M. M.; Gege, P.; Simis, S. G. H.; Eleveld, M. A.; Peters, S. W. M. Validation of a Spectral Correction Procedure for Sun and Sky Reflections in Above-Water Reflectance Measurements. *Opt. Express* **2017**, *25* (16), A742, DOI: 10.1364/oe.25.00a742
